# Rv0004 is a new essential member of the mycobacterial DNA replication machinery

**DOI:** 10.1371/journal.pgen.1007115

**Published:** 2017-11-27

**Authors:** Katherine M. Mann, Deborah L. Huang, Anna J. Hooppaw, Michelle M. Logsdon, Kirill Richardson, Hark Joon Lee, Jacqueline M. Kimmey, Bree B. Aldridge, Christina L. Stallings

**Affiliations:** 1 Department of Molecular Microbiology, Washington University School of Medicine, St. Louis, Missouri, United States of America; 2 Department of Molecular Biology and Microbiology, Tufts University School of Medicine, Boston, Massachusetts, United States of America; 3 Department of Biomedical Engineering, Tufts University School of Engineering, Medford, Massachusetts, United States of America; University of Washington School of Medicine, UNITED STATES

## Abstract

DNA replication is fundamental for life, yet a detailed understanding of bacterial DNA replication is limited outside the organisms *Escherichia coli* and *Bacillus subtilis*. Many bacteria, including mycobacteria, encode no identified homologs of helicase loaders or regulators of the initiator protein DnaA, despite these factors being essential for DNA replication in *E*. *coli* and *B*. *subtilis*. In this study we discover that a previously uncharacterized protein, Rv0004, from the human pathogen *Mycobacterium tuberculosis* is essential for bacterial viability and that depletion of *Rv0004* leads to a block in cell cycle progression. Using a combination of genetic and biochemical approaches, we found that Rv0004 has a role in DNA replication, interacts with DNA and the replicative helicase DnaB, and affects DnaB-DnaA complex formation. We also identify a conserved domain in Rv0004 that is predicted to structurally resemble the N-terminal protein-protein interaction domain of DnaA. Mutation of a single conserved tryptophan within Rv0004’s DnaA N-terminal-like domain leads to phenotypes similar to those observed upon *Rv0004* depletion and can affect the association of Rv0004 with DnaB. In addition, using live cell imaging during depletion of *Rv0004*, we have uncovered a previously unappreciated role for DNA replication in coordinating mycobacterial cell division and cell size. Together, our data support that *Rv0004* encodes a homolog of the recently identified DciA family of proteins found in most bacteria that lack the DnaC-DnaI helicase loaders in *E*. *coli* and *B*. *subtilis*. Therefore, the mechanisms of Rv0004 elucidated here likely apply to other DciA homologs and reveal insight into the diversity of bacterial strategies in even the most conserved biological processes.

## Author summary

DNA is the molecule that encodes all of the genetic information of an organism. In order to pass genes onto the next generation, DNA has to first be copied through a process called DNA replication. Most of the initial studies on bacterial DNA replication were performed in *Escherichia coli* and *Bacillus subtilis*. While these studies were very informative, there is an increasing appreciation that more distantly related bacteria have diverged from these organisms in even the most fundamental processes. Mycobacteria, a group of bacteria that includes the human pathogen *Mycobacterium tuberculosis*, are distantly related to *E*. *coli* and *B*. *subtilis* and lack some of the proteins used for DNA replication in those model organisms. In this study, we discover that a previously uncharacterized protein in Mycobacteria, named Rv0004, is essential for bacterial viability and involved in DNA replication. *Rv0004* is conserved in most bacteria but is absent from *E*. *coli* and *B*. *subtilis*. Since *Rv0004* is essential for mycobacterial viability, this study both identifies a future target for antibiotic therapy and expands our knowledge on the diversity of bacterial DNA replication strategies, which may be applicable to other organisms.

## Introduction

The ability to maintain, replicate, and express genetic information encoded in DNA is critical to all domains of life. DNA replication studies in *Escherichia coli* and *Bacillus subtilis* have elucidated the mechanisms of bacterial DNA replication initiation, elongation, and termination, but the applicability of many of these findings to other bacteria is less clear. Briefly, initiation begins when DnaA, the initiator protein, binds to specific sites located at the origin of replication (*oriC*) and oligomerizes, forming a nucleoprotein complex that results in the melting of the adjacent DNA [1]. Next, helicase loaders and accessory primosomal proteins, with the help of DnaA, load the replicative helicase onto melted DNA [2,3]. The replicative helicase then binds the primase, which lays down short RNA primers [2]. Clamp loader complexes load DNA Polymerase III (Pol III) onto primed DNA, allowing replication elongation to begin [1]. Elongation proceeds bi-directionally from *oriC* until it reaches termination sites bound by terminator proteins [4].

Although the general stages of DNA replication are likely conserved in all bacteria, many steps have not been studied outside of the model organisms *E*. *coli* and *B*. *subtilis*. In particular, DNA replication is not well understood in mycobacteria, including the human pathogen *Mycobacterium tuberculosis* (*Mtb*). While high-fidelity DNA replication and repair is critical to maintain chromosomal integrity, mutations generated by error-prone DNA replication can enhance *Mtb* virulence and lead to antibiotic resistance [5]. The study of DNA replication and repair in mycobacteria is particularly relevant given that all acquired drug resistance in *Mtb* arises through chromosomally encoded mutations [6,7]. Mycobacteria encode homologs of some, but not all, DNA replication proteins and it is not clear how most mycobacterial homologs function relative to their *E*. *coli* counterparts. For example, Rock *et al*. recently showed that the DNA Pol III ε exonuclease, which is essential for replication fidelity in *E*. *coli*, is dispensable in *Mtb* [8]. There are also a number of processes essential for efficient DNA replication in *E*. *coli* and *B*. *subtilis* for which homologs have not been identified in mycobacteria, including regulators of DnaA activity (Hda in *E*. *coli*, YabA in *B*. *subtilis* [9]), proteins that load the replicative helicase (DnaC in *E*. *coli*, DnaI in *B*. *subtilis* [2,10]), and replication terminator proteins (Tus in *E*. *coli* and RTP in *B*. *subtilis* [11]). The proteins required for these processes in *E*. *coli* and *B*. *subtilis* are functionally analogous but are not conserved in sequence. Therefore, functionally similar mycobacterial proteins could exist and remain unidentified due to sequence divergence.

In this study we have discovered that *Rv0004* in *Mtb* and *MSMEG_0004* in *Mycobacterium smegmatis* are essential for DNA replication even though they are absent from *E*. *coli* and *B*. *subtilis*, the organisms traditionally used to study bacterial DNA replication. Rv0004 had never before been studied but is predicted to contain a domain of unknown function 721 (DUF721, PF05258). In a recent publication, Brezellec *et al*. used bioinformatics to identify DUF721-containing proteins in 23 out of 26 bacterial phyla and named the members of this protein family “DciA,” for DnaC DnaI antecedent, based on the finding that these proteins preceded the DnaC-DnaI helicase loading systems of *E*. *coli* and *B*. *subtilis* [12]. While Brezellec *et al*. illustrate how widely distributed DciA homologs are, which underscores the importance of work on DciA proteins to the field of bacterial DNA replication, the experimental data is limited to showing that *Pseudomonas aeruginosa* DciA is important for DNA replication in *P*. *aeruginosa* and associates with DnaB in a bacterial two-hybrid assay [12]. Therefore, a molecular and biochemical analysis of DciA has yet to be performed and is necessary to define the basis of DciA’s interaction with DnaB outside of the bacterial two-hybrid system, to determine how DciA’s association with DnaB relates to its role in DNA replication, and to elucidate other activities for DciA in the cell.

In this manuscript we perform the first mechanistic studies on a DciA homolog and show that *Mtb* Rv0004 (DciA_Mtb_) directly binds DNA and the replicative helicase DnaB to regulate the interaction of DnaB with the initiator protein DnaA. We discover that the DUF721 in DciA proteins comprises a protein-protein interaction domain that is predicted to structurally resemble the N-terminus of DnaA. We provide data to support the importance of this domain by showing that the mutation of a single conserved tryptophan within DciA_Mtb_’s DnaA N-terminal-like domain leads to defects in DciA_Mtb_’s cellular activity and can affect the association of DciA_Mtb_ with DnaB. In addition, using live cell imaging during depletion of *dciA*_*Mtb*_ we have uncovered a previously unappreciated role for DNA replication in the coordination of mycobacterial cell division and cell size. Together, these studies elucidate a mechanism by which DciA proteins affect DNA replication initiation, identify a function for a conserved protein domain, and provide insight into the influence of DNA replication on cell cycle in mycobacteria.

## Results

### Identification of *MSMEG_0004* and *Rv0004*

**Fig 1:**
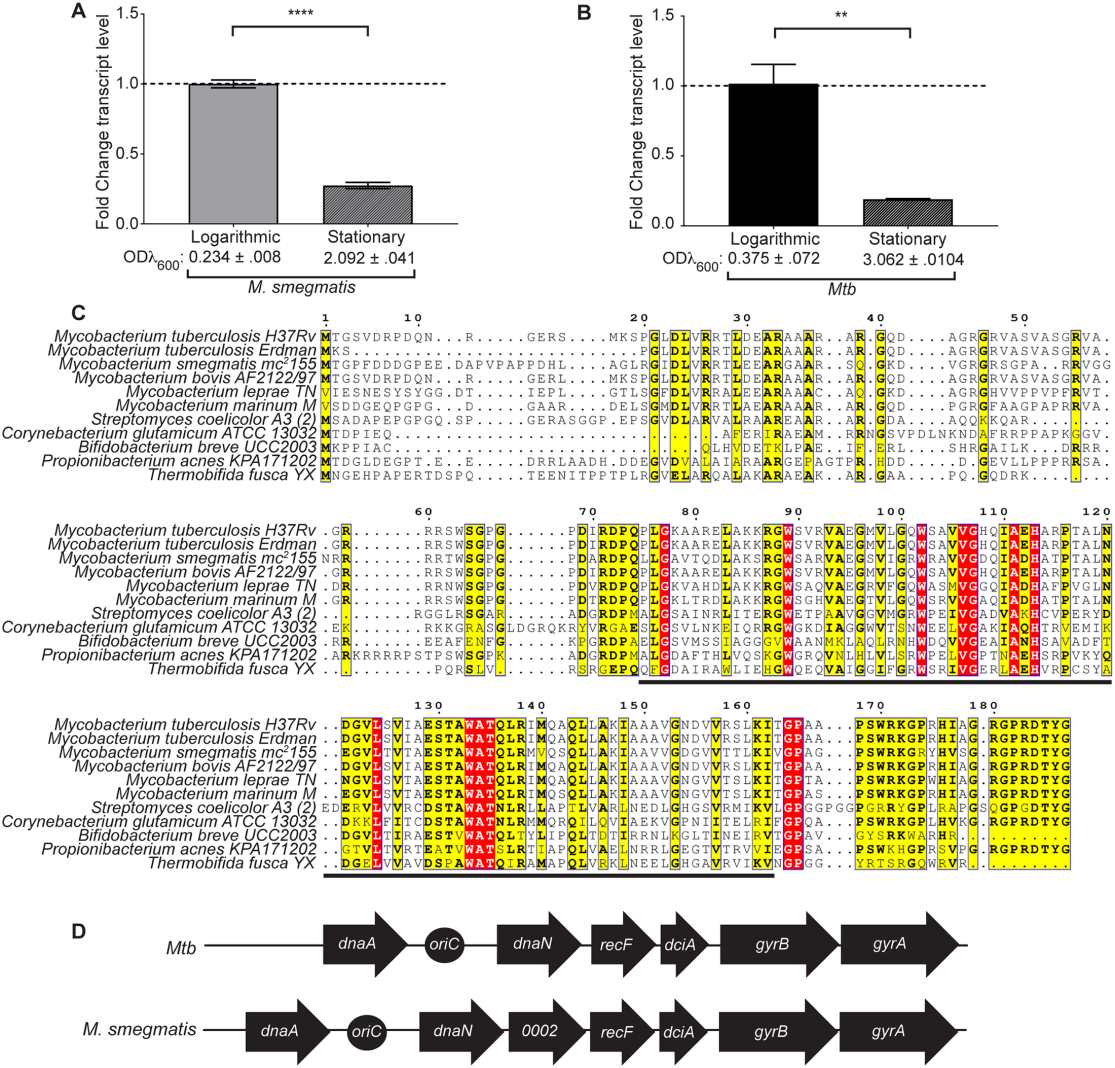
*0004* (*dciA*) is regulated by growth phase and is conserved throughout Actinobacteria. (A,B) Fold change in *MSMEG*_*0004* (*dciA*_*Msm*_*)* transcript in *M*. *smegmatis* (A) and *Rv0004* (*dciA*_*Mtb*_*)* transcript in *Mtb* (B) normalized to 16S rRNA in stationary phase versus logarithmic (log) phase, log phase levels were set to 1. Mean ODλ_600_ ± SD is indicated. Bars represent means ± SEM (n = 3). **** p <0.0001, ** p <0.01, statistical significance was determined by Student’s unpaired *t*-test. (C) Multiple sequence alignment of 0004 (DciA) protein sequences from different Actinobacteria. Red residues are identical across all sequences, while yellow residues have global similarity scores of at least 0.7 calculated by MultAlin. Within yellow residues, bold letters signify the most common residue. The black line indicates the region of the protein that constitutes DUF721. (D) Genomic context of the *dciA (0004)* gene in *M*. *smegmatis* and *Mtb*.

In previous work we probed the transcriptional responses of *M*. *smegmatis*, a non-pathogenic model organism for *Mtb*, to double-stranded DNA (dsDNA) breaks [13]. We identified *MSMEG_0004* as being upregulated in response to dsDNA breaks that occurred during logarithmic (log) but not stationary phase. By measuring the expression of *MSMEG_0004* during log versus stationary growth phase in the absence of induced DNA damage, we found that *MSMEG_0004* is also more highly expressed in log phase in the absence of stress (Fig 1A). We observed a similar result for the *Mtb* homolog *Rv0004* (Fig 1B). Together, these data suggest that expression of *0004* genes is important while the bacteria are actively growing and dividing.

*MSMEG_0004* homologs, which are not present in eukaryotes, *E*. *coli*, or *B*. *subtilis*, encode hypothetical proteins that contain a domain of unknown function (DUF721; PFAM05258, Fig 1C) and are predicted to be nucleic acid-binding proteins (COG5512 family members) [14]. Brezellec *et al*. recently identified DUF721 as being widely conserved in proteins they termed DciA [12]. Based on the presence of DUF721 in Rv0004 and MSMEG_0004, we will refer to *Rv0004* as *dciA*_*Mtb*_ and *MSMEG_0004* as *dciA*_*Msm*_.

*dciA*_*Msm*_ is located in an operon next to *oriC* that also contains *dnaN* and *recF*, which encode the DNA Pol III beta clamp and a DNA repair protein, respectively (Fig 1D) [15,16]. This operon is located between *dnaA* and the *gyrB-gyrA* operon, which encode the replication initiator protein and bacterial gyrase, respectively. This genome structure is conserved between *M*. *smegmatis* and *Mtb* except for *MSMEG_0002*, a gene that is predicted to encode a 6-phosphogluconate dehydrogenase and is encoded at a separate genomic location in *Mtb* (Fig 1D) [17]. The orientation of the DNA replication and repair genes *dnaA*, *dnaN*, *recF*, *gyrB*, *gyrA* near *oriC* is a conserved feature in many bacteria [18]. Like *dciA*_*Msm*_, *dnaA*, *dnaN*, and *recF* were all more highly expressed in log phase versus stationary phase, consistent with roles in DNA replication (S1 Fig). The genomic location and the increased expression of *dciA*_*Mtb*_ and *dciA*_*Msm*_ during log phase support a link between the mycobacterial DciA proteins and DNA replication or repair.

### *dciA* is essential for *M*. *smegmatis* viability in culture

**Fig 2:**
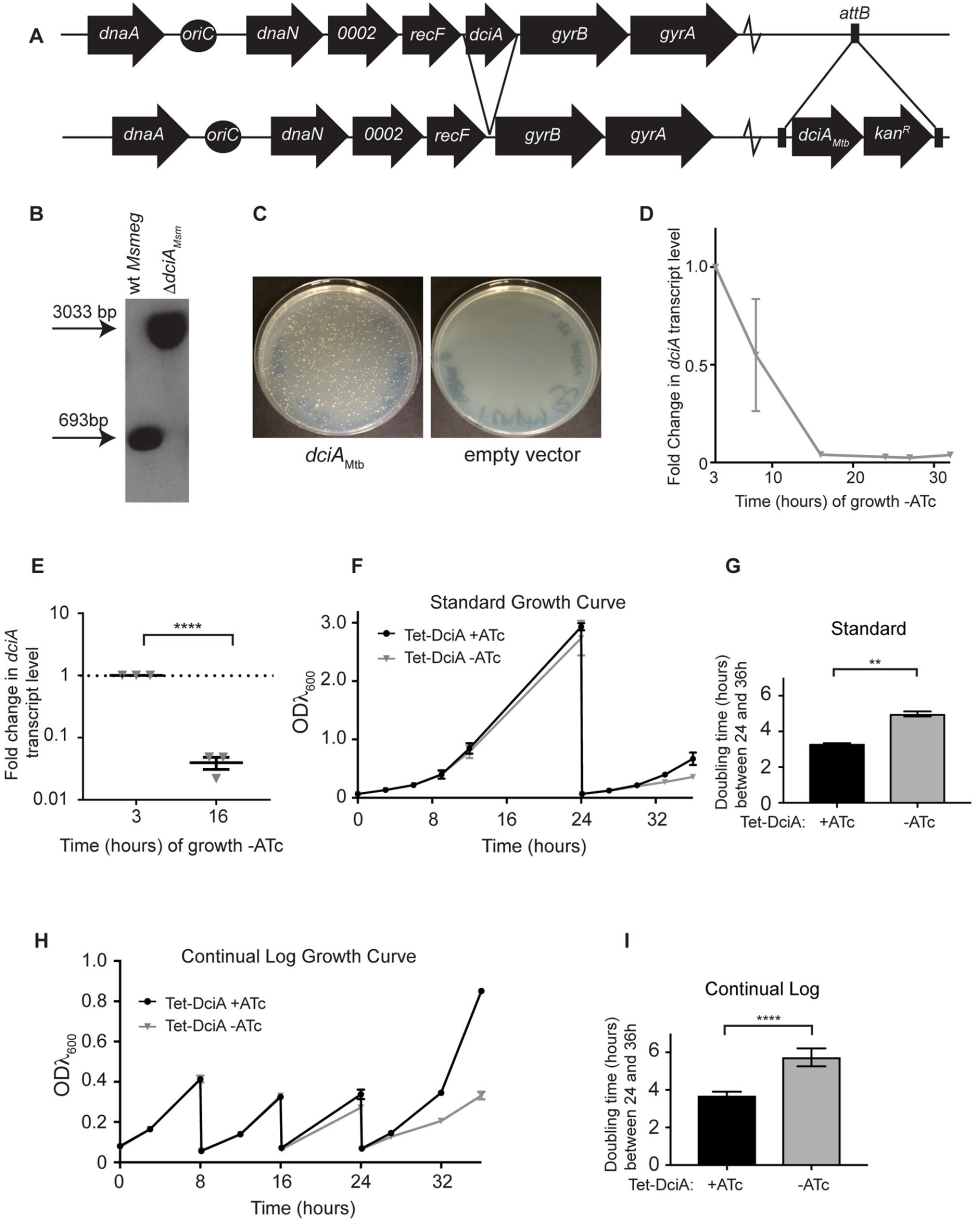
*dciA* is essential for growth in *M*. *smegmatis*. (A) Construction of Δ*dciA*_*Msm*_ *attB*::tet*dciA*_*Mtb*_. (B) Southern blot of wild-type *M*. *smegmatis* (wt *Msmeg*) and Δ*dciA*_*Msm*_ *attB*::tet*dciA*_*Mtb*_ genomic DNA. After digestion with PvuII, wt *Msmeg* yields a 693 bp band and Δ*dciA*_*Msm*_ yields a 3033bp band. (C) Agar plates 3 days after transformation of Δ*dciA*_*Msm*_ *attB*::tet*dciA*_*Mtb*_ with a gene-switching, *attB*-integrating plasmid encoding either *dciA*_*Mtb*_ (left) or not (right). After incubation of the empty vector plate for over 10 days, a *dciA* null strain was never recovered. This experiment was repeated at least five times. (D,E) Fold change in *dciA*_*Mtb*_ transcript normalized to *sigA* during Tet-DciA growth in the absence of ATc where the level at 3 hours (h) for each culture is set to 1. (D) Time course from Tet-DciA cultures grown -ATc, with each point representing n = 4 except the 16 h and 27 h points, which each represent n = 3. (E) *dciA*_*Mtb*_ transcript levels at 16 h growth -ATc compared to 3 h. Center values and error bars represent mean ± SEM. The fold change of 0.040 ± 0.009 is equivalent to a 96.0% ± 0.9% reduction in transcript at 16 h. (F) Representative standard growth curve of Tet-DciA grown +ATc (n = 3) or -ATc (n = 3). Standard growth curves were performed four times. (G) Doubling times from 24 h to 36 h of standard growth curves calculated using the exponential growth equation. Bars indicate means ± SEM (Tet-DciA +ATc n = 6; Tet-DciA -ATc n = 9). (H) Representative continual log growth curve of Tet-DciA grown +ATc (n = 3) or -ATc (n = 3). Continual growth curves were repeated eight times. (I) Doubling times from 24 h to 36 h of continual log growth curve calculated using the exponential growth equation. Bars indicate means ± SEM (Tet-DciA +ATc n = 18; Tet-DciA -ATc n = 21). **** p <0.0001, ** p <0.01, statistical significance determined by Student’s unpaired *t*-test.

In order to study the roles for *dciA*_*Msm*_ and *dciA*_*Mtb*_ in mycobacteria, we took a reverse genetic approach. Attempts to delete *dciA*_*Msm*_ from *M*. *smegmatis* and *dciA*_*Mtb*_ from *Mtb* were unsuccessful, suggesting these genes are essential for viability. To study the *dciA* genes, we constructed a merodiploid strain in which the *dciA*_*Mtb*_ gene was integrated at the *M*. *smegmatis attB* site under the control of a promoter that contains tet operator sites and is linked to a kanamycin resistance cassette (Fig 2A, S1 and S2 Tables). We then deleted *dciA*_*Msm*_ from its endogenous locus (Fig 2B), resulting in the strain Δ*dciA*_*Msm*_ *attB*::tet*dciA*_*Mtb*_ (Fig 2A). We used the Δ*dciA*_*Msm*_ *attB*::tet*dciA*_*Mtb*_ strain to study *dciA*_*Mtb*_ in the context of *M*. *smegmatis*. Unfortunately, we were unable to engineer similar strains in *Mtb*, likely due to the difficulty of manipulating the genome near *oriC*. To confirm that *dciA* is essential in *M*. *smegmatis*, we used a gene-switching technique [19] to replace the kanamycin resistance cassette-linked *dciA*_*Mtb*_ allele at the *attB* site in the Δ*dciA*_*Msm*_ *attB*::tet*dciA*_*Mtb*_ strain with a zeocin-resistant plasmid that was either empty or expressed *dciA*_*Mtb*_. We were able to switch in the zeocin-resistant plasmid expressing *dciA*_*Mtb*_ but we were unable to recover a *dciA* gene null mutant (Fig 2C), further supporting that *dciA*_*Msm*_ is essential for viability.

### Depletion of *dciA*_*Mtb*_ results in inhibition of growth

To study the loss of *dciA* expression in *M*. *smegmatis*, the Δ*dciA*_*Msm*_ *attB*::tet*dciA*_*Mtb*_ strain was transformed with an episomal plasmid expressing a Tet-ON repressor (TetR) [20] (S1 and S2 Tables). In this *M*. *smegmatis* Δ*dciA*_*Msm*_ *attB*::tet*dciA*_*Mtb*_ +pTetR strain, referred to as Tet-DciA, *dciA*_*Mtb*_ transcript is only expressed in the presence of anhydrotetracycline (ATc). We monitored *dciA*_*Mtb*_ depletion by diluting cultures of Tet-DciA grown in the presence of ATc (+ATc) into liquid media lacking ATc (-ATc) and collecting RNA at 3, 8, 16, 24, 28, and 32 hours after growth -ATc. After 16 hours in depleting conditions, *dciA*_*Mtb*_ transcript levels were 96% lower than at 3 hours (Fig 2D and 2E). To characterize the requirement of *dciA*_*Mtb*_ expression for growth, we monitored the growth of the Tet-DciA strain during *dciA*_*Mtb*_ transcript depletion. Over the first 24 hours, there was no significant difference in the growth of Tet-DciA in depleting (-ATc) or replete (+ATc) conditions (Fig 2F). However, after diluting the stationary phase culture back to early log phase at 24 hours, depleted cells grew more slowly than controls (Fig 2F). We calculated the doubling times of Tet-DciA cultured ± ATc and found that after 24 hours, *M*. *smegmatis* depleted of *dciA*_*Mtb*_ grew significantly more slowly than the replete controls (5.8 hour versus 3.7 hour doubling time) (Fig 2G).

To determine whether the growth defect of *dciA*_*Mtb*_-depleted cells was due to an inability to recover from stationary phase versus DciA_Mtb_ playing a critical role in growth during log phase, we performed a continual log liquid growth curve where cultures were diluted to early log phase every 8 hours to ensure that they did not enter stationary phase. Similar to the standard growth curve experiments, *M*. *smegmatis* depleted of *dciA*_*Mtb*_ did not show a significant growth defect until after 24 hours of growth–ATc (Fig 2H). We calculated the doubling times of Tet-DciA cultured ± ATc and found that after 24 hours of continual log growth, *M*. *smegmatis* depleted of *dciA*_*Mtb*_ grew significantly more slowly than the replete controls (4.7 hour versus 3.4 hour doubling time) (Fig 2I). After 40 hours of growth in depleting conditions, suppressors of the Tet-ON system are selected for and the levels of *dciA*_*Mtb*_ transcript are no longer controlled by ATc. Together, these data demonstrate that DciA is important for *M*. *smegmatis* growth.

### Depletion of *dciA*_*Mtb*_ results in a block in cell cycle progression

**Fig 3:**
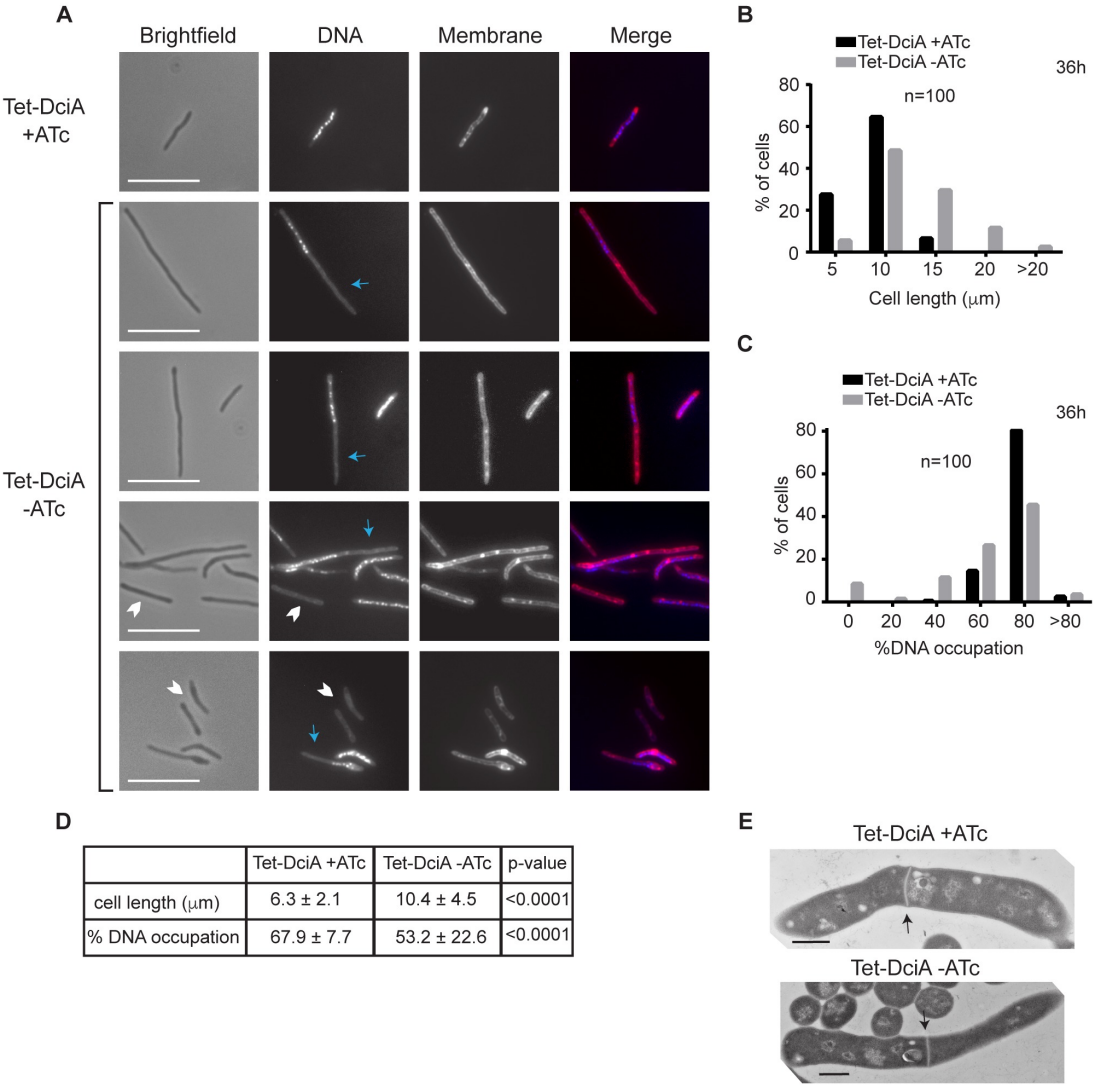
Depletion of DciA leads to a block in cell cycle progression. (A) Fluorescence microscopy of Tet-DciA cells grown for 36 hours (h) +ATc (top row) or -ATc (bottom four rows) pictured in brightfield, stained with DAPI for DNA, or stained with FM1-43FX for membranes. Blue arrows indicate areas free of DNA staining and white chevrons indicate anucleate cells. Scale bars are 10 μm. (B) Cell lengths of Tet-DciA grown ± ATc at 36 h. (C) % DNA occupation, which is the nucleoid length divided by the cell length times 100, of Tet-DciA grown ± ATc at 36 h. (D) Means ± SD from data in (B) and (C). (E) TEM of Tet-DciA grown ± ATc at 36 h. Arrows indicate septa. Scale bars are 500 nm.

We have shown that *dciA* is essential for growth in culture and is involved in responding to DNA damage in *M*. *smegmatis*. However, a role in DNA damage responses alone cannot explain the essentiality of *dciA*, since genes key for mycobacterial DNA repair pathways are not essential *in vitro* [21–24]. The location of mycobacterial *dciA* genes in an operon with and adjacent to genes involved in DNA replication raises the question of whether DciA plays a role in this essential process in mycobacteria. In addition, *P*. *aeruginosa* DciA (DciA_Pa_) has been implicated in DNA replication [12]. To investigate a role for mycobacterial DciA in DNA replication and the bacterial cell cycle, we monitored the cellular morphology of Tet-DciA cultured ± ATc in the continual log growth curve (Fig 2H). By 24 hours of depletion, Tet-DciA cultured without ATc were on average 12.9% (p <0.01) longer than Tet-DciA cultured in *dciA*_*Mtb*_-replete conditions (S2 Fig). After 36 hours, Tet-DciA cultured without ATc averaged over 60% longer (p <0.0001) than Tet-DciA grown in *dciA*_*Mtb*_-replete conditions (Fig 3A, 3B and 3D). The elongated cellular morphology observed during *dciA*_*Mtb*_ depletion indicates a block in cell cycle progression.

To characterize where in the cell cycle *dciA*_*Mtb*_-depleted *M*. *smegmatis* is blocked, we analyzed nucleoid morphology and septum formation. To observe nucleoid morphology, Tet-DciA was grown ± ATc, DNA was stained with DAPI, and cells were visualized by fluorescent microscopy (Fig 3A). The nucleoid in *dciA*_*Mtb*_-replete Tet-DciA (+ATc) appears as several distinct puncta throughout the length of the cell (Fig 3A, top row, second panel), as has been reported previously for *M*. *smegmatis* [25]. Following *dciA*_*Mtb*_ depletion, the DAPI-stained nucleoid still appeared as several distinct puncta, but was not distributed throughout the length of the cell. Instead, there were areas free of DNA staining found at the poles (Fig 3A). We quantified the abnormal nucleoid morphology using “% DNA occupation,” which represents the nucleoid length as a percentage of the total cell length. Tet-DciA depleted for *dciA*_*Mtb*_ exhibit significantly lower % DNA occupation starting at 16 hours of depletion (Fig 3C and 3D; S2 Fig). The observation that depletion of *dciA*_*Mtb*_ leads to significantly lower % DNA occupation at 16 hours even though cell lengths are not significantly longer at this time point (S2 Fig) indicates that abnormal nucleoid morphology is the earlier phenotype, and that a DNA-related function causes the cell cycle block and slowed growth (Fig 2I).

We also observed the presence of 9% anucleate cells by DAPI staining upon *dciA*_*Mtb*_ depletion (Fig 3A). Anucleate cells result when DNA replication and cellular division are uncoupled. The presence of anucleate cells also indicates that cell division is still able to occur. To confirm that septum formation was intact, we used transmission electron microscopy (TEM) and found that cells depleted for *dciA*_*Mtb*_ were still able to form normal septa (Fig 3E).

To visualize cell division of *dciA*_*Mtb*_-depleted *M*. *smegmatis* in real time, we performed live-cell imaging [26] of Tet-DciA grown ± ATc in a constant-flow microfluidic device (S1 and S2 Movies). Using FM4-64 membrane stain, we observed that *dciA*_*Mtb*_-depleted cells can form septa and undergo cell division (S2 Movie). We also detected increased cell death in *dciA*_*Mtb*_-depleted cells compared to controls, where dead cells stop elongating and take up more FM4-64 dye, thus exhibiting a rapid increase in fluorescence (S2 Movie). 9.4% of *dciA*_*Mtb*_-depleted cells displayed these cell death characteristics. In support of our fixed fluorescent microscopy, Tet-DciA cells grown -ATc were on average 56% longer at birth and division than Tet-DciA grown +ATc (S3 Fig, S1 and S2 Movies, p <0.0001). However, despite the increased average length of *dciA*_*Mtb*_-depleted cells, we also observed division of unusually small cells (S3E Fig), many of which died shortly after division. In general, we observed greater variability in birth length in *dciA*_*Mtb*_ depleted versus replete cells, with coefficients of variation of 41.7% and 18.2%, respectively (S3C Fig). The increased heterogeneity among cell birth lengths during *dciA*_*Mtb*_ depletion indicates a disruption in the coordination of septum formation with cell growth, leading to the dysregulation of cell size. Thus, time-lapse imaging demonstrates that in addition to *dciA*_*Mtb*_-depleted cells being elongated on average, they are also characterized by increased variation in cell size and frequency of death.

**Fig 4:**
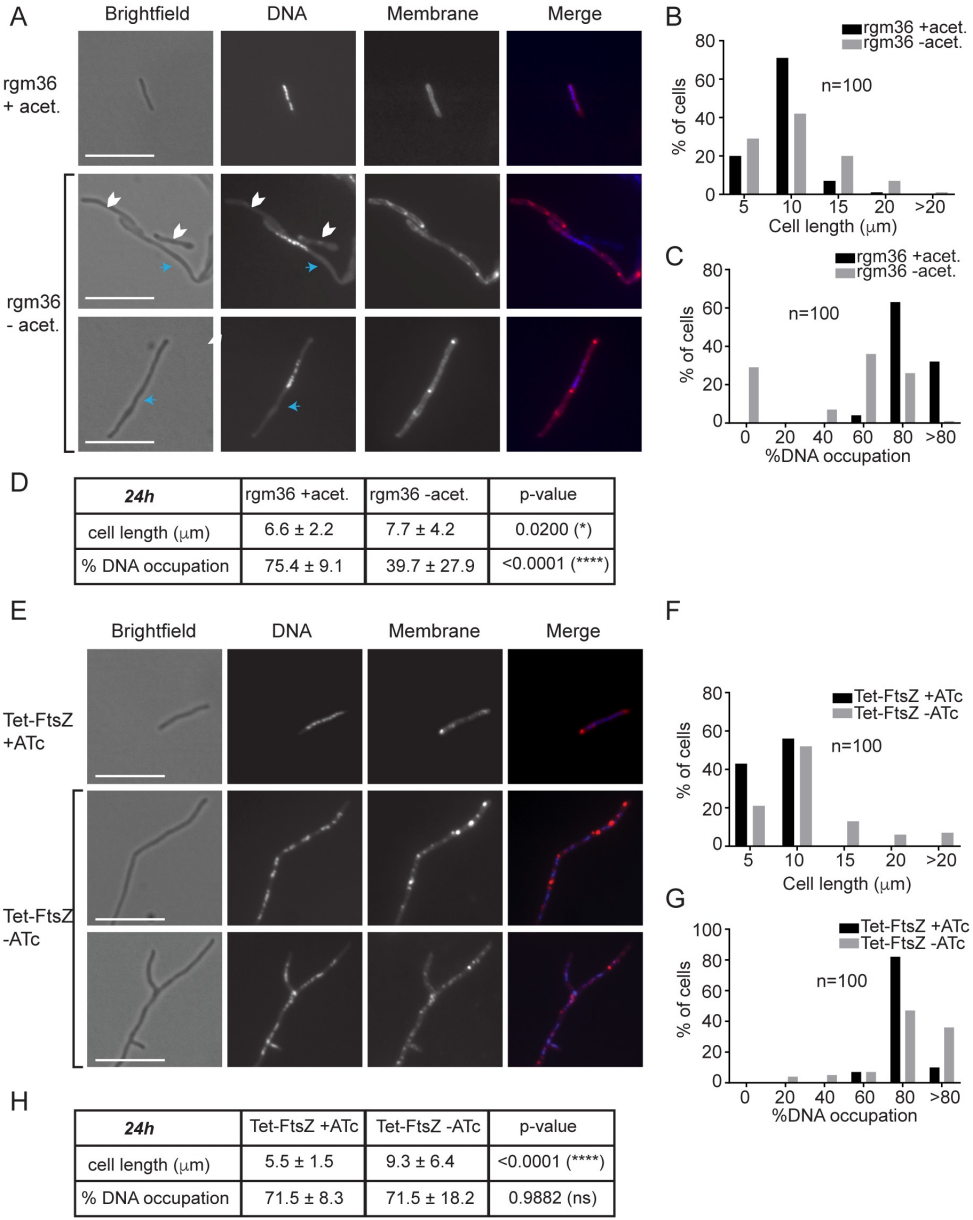
*dciA*_*Mtb*_ depletion phenocopies *dnaA* depletion and not *ftsZ* depletion in *M*. *smegmatis*. (A) Fluorescence microscopy of rgm36, an acetamide-inducible *dnaA* depletion *M*. *smegmatis* strain [25], grown in the presence (top row) or absence (bottom two rows) of the acetamide (acet.) inducer pictured in brightfield, stained with DAPI for DNA, and stained with FM1-43FX for membrane. Blue arrows indicate areas free of DNA staining, while white chevrons indicate anucleate cells. Scale bars are 10 μm. (B) Cell length and (C) DNA occupation histograms of rgm36 cells after 24 hours of growth in depleting (-acet.) or replete (+acet.) conditions. (D) Table displays averages ± standard deviations from data depicted in histograms (B,C), along with p-values determined by Student’s *t*-test. (E) Fluorescence microscopy of csm362, a TetOn FtsZ depletion *M*. *smegmatis* strain, in the presence (top row) or absence (bottom two rows) of ATc pictured in brightfield, stained with DAPI for DNA, and stained with FM1-43FX for membrane. Scale bars are 10 μm. (F) Cell length and (G) % DNA occupation histograms of csm362 cells after 24 hours of growth in depleting (-ATc) or replete (+ATc) conditions. (H) Table displays averages ± standard deviations from data depicted in histograms (F,G), along with p-values determined by Student’s *t*-test.

Together, our data show that cell division is intact but chromosome replication or segregation is blocked during *dciA*_*Mtb*_ depletion. In support of this conclusion, the abnormal nucleoid morphologies observed in *M*. *smegmatis* depleted of *dciA*_*Mtb*_ phenocopy those of *M*. *smegmatis* depleted of DnaA, the chromosomal replication initiator protein [25] (Fig 4A–4D, S4 Fig), but not *M*. *smegmatis* depleted of FtsZ, the protein that comprises the Z-ring precursor of the septum [27] (Fig 4E–4H, S5 Fig).

### *dciA* depletion results in decreased DNA synthesis

**Fig 5:**
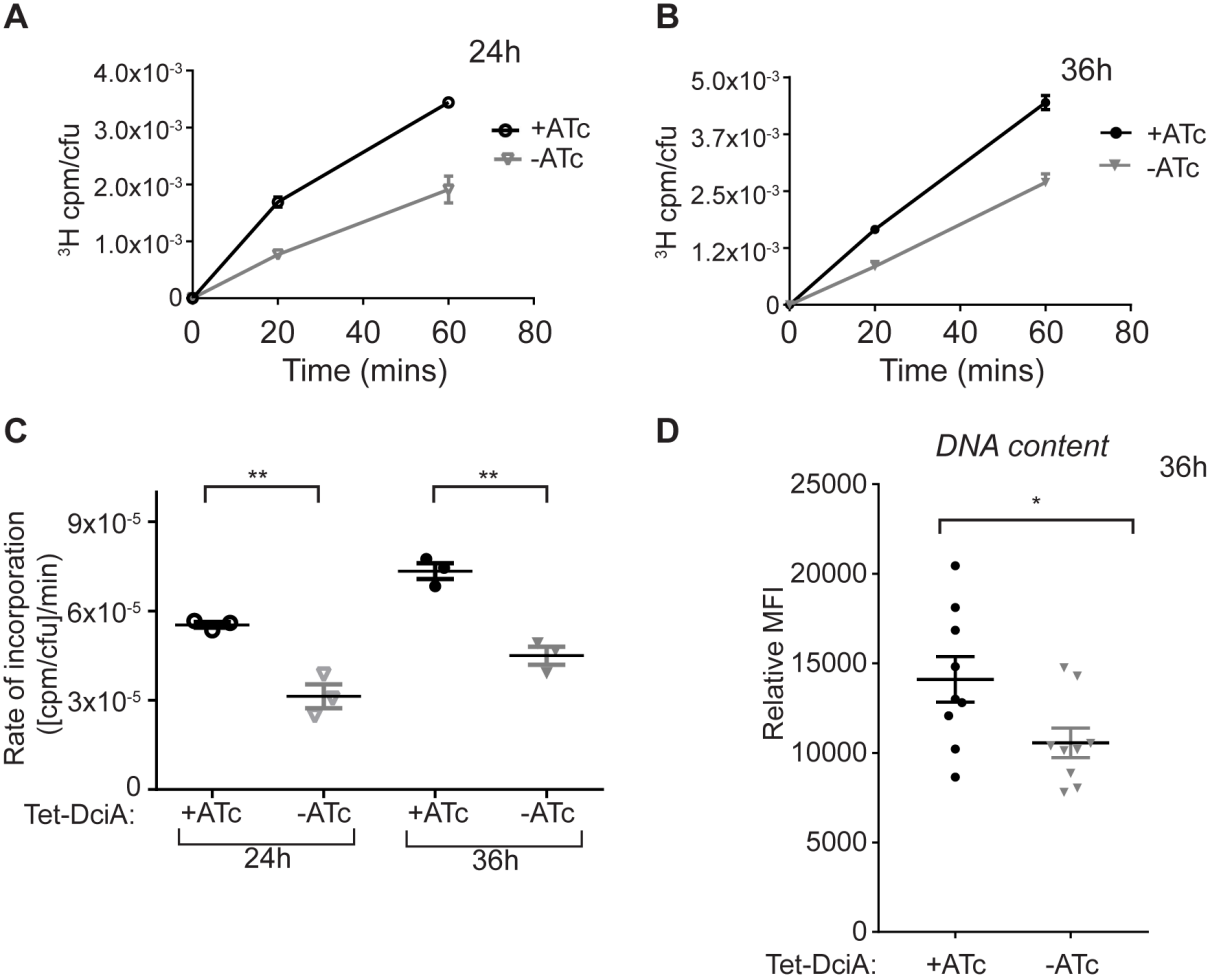
Depletion of DciA leads to decreased DNA synthesis. (A,B) ^3^H incorporation in Tet-DciA as measured by ^3^H counts per minute (cpm) per colony forming unit (cfu) at 24 (A) and 36 (B) hours (h) growth ± ATc. Symbols represent mean ± SEM (n = 3). (C) Rates of ^3^H incorporation calculated from slopes of the lines of the linear regression from (A) and (B), symbols represent each biological replicate. (D) Geometric mean fluorescence intensity (MFI) of DAPI stained Tet-DciA grown ± ATc for 36 h relative to an unstained sample determined by flow cytometry. Representative flow cytometry histograms are found in S6A Fig. Symbols represent each biological replicate, center values and error bars represent means ± SEM. ** p <0.01, * p <0.05, statistical significance determined by Student’s unpaired *t*-test.

The data so far support a model that DciA homologs are required for either DNA replication or chromosome segregation. To determine if *dciA*_*Mtb*_-depleted *M*. *smegmatis* is defective in DNA replication, we directly measured rates of DNA synthesis using a nucleotide incorporation assay [25,28]. Specifically, we determined the rates of [5,6-^3^H]-thymidine incorporation into DNA by Tet-DciA cells grown ± ATc in continual log phase. We found that the rate of DNA synthesis in *M*. *smegmatis* was significantly lower at 24 hours (Fig 5A and 5C) and 36 hours (Fig 5B and 5C) in Tet-DciA grown -ATc relative to Tet-DciA grown +ATc, proving that the rate of DNA replication itself decreases upon *dciA*_*Mtb*_ depletion. To further confirm this defect in DNA replication, we stained Tet-DciA grown ± ATc with DAPI and measured DNA content using flow cytometry. *dciA*_*Mtb*_-depleted cells had lower DNA content per cell based on the mean fluorescence intensity (MFI) of DAPI staining relative to controls (Fig 5D and S6A Fig). Together, these data demonstrate that DciA_Mtb_ is involved in DNA replication, but do not differentiate between which step(s) of DNA replication DciA_Mtb_ acts.

### DciA_Mtb_ binds DNA

**Fig 6:**
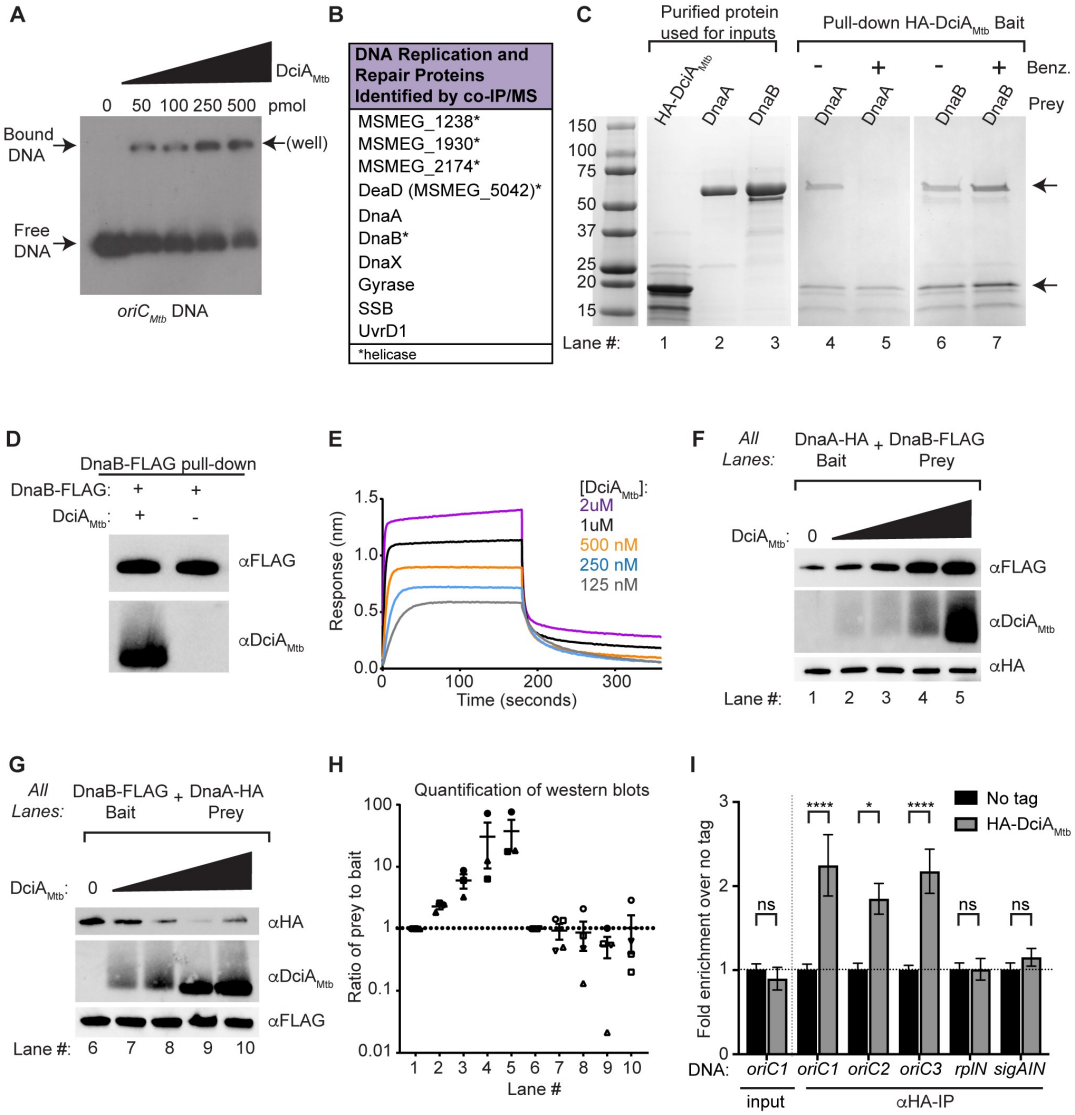
DciA_Mtb_ binds DNA, interacts directly with DnaB, and affects the DnaB-DnaA interaction. (A) Autoradiograph of EMSA with DciA_Mtb_ protein and 4.8ng *oriC*_*Mtb*_ dsDNA separated by native PAGE. All lanes contain ^32^P-labeled *oriC*_*Mtb*_ DNA. Amount of DciA_Mtb_ in each lane is indicated. (B) Proteins involved in DNA replication or repair that were identified by MS analysis as co-immunoprecipitating with HA-DciA_Mtb_. (C) GelCode Blue-stained SDS-PAGE of pull-down experiments using HA-DciA_Mtb_, DnaB, and DnaA. Lanes 1–3 after ladder contain purified proteins used in pull-downs. Lanes 4–7 contain eluates from pull downs with immobilized HA-DciA_Mtb_ incubated with DnaA (lanes 4–5) or DnaB (lanes 6–7). Proteins used were purified in the absence (lanes 4,6) or presence (lanes 5,7) of Benzonase (Benz.). Top arrow indicates the size of DnaA and DnaB. Bottom arrow indicates the size of HA-DciA_Mtb_. (D) Western blot analysis of pull-downs with DnaB-FLAG as bait and DciA_Mtb_ as prey. (E) Representative curves from BLI showing the association and dissociation of DciA_Mtb_ at the indicated concentrations with immobilized biotinylated-DnaB. (F,G) Representative western blot of pull-downs with (F) DnaA-HA as bait and DnaB-FLAG as prey or (G) DnaB-FLAG as bait and DnaA-HA as prey and either no (lanes 1,6), 0.5x (lanes 2,7), 1x (lanes 3,8), 2x (lanes 4,9), or 4x (lanes 5,10) molar DciA_Mtb_ relative to the bait. (H) Quantification of the ratio of prey-to-bait for triplicate western blots like those shown in (F) and (G) where the ratio for lanes with no DciA_Mtb_ is set to 1 and the ratio for all other samples is relative to the lane with no DciA_Mtb_. Symbols represent each replicate, center values and error bars represent mean ± SEM. (I) Fold enrichment in levels of DNA fragments containing *oriC* (*oriC1-3*, see S8A Fig), the promoter of the *rplN* operon (*rplN*), or the *sigA* coding sequence (*sigAIN*) in samples immunoprecipitated with an anti-HA antibody (αHA-IP) or input samples (input) from strains expressing untagged DciA_Mtb_ (No tag, black bars) and HA-DciA_Mtb_ (grey bars) strains. Data is represented as fold enrichment relative to No tag levels. Bars represent mean ± SEM (n = 5 except No tag *sigAIN*, which is n = 4). **** p <0.0001, * p <0.05, ns is not significant, statistical significance was determined by one-way ANOVA and Tukey’s multiple comparison test.

To determine how DciA proteins function in DNA replication, we investigated macromolecular interaction partners of DciA_Mtb_. DciA_Mtb_ has a calculated isoelectric point (pI) around 12, indicating that the protein is positively-charged at neutral pH. Other proteins in mycobacteria with high isoelectric points include histone-like proteins (H-NS, HupB) and integration host factor (IHF), which are all known to bind DNA [29–32]. DciA_Mtb_, as a member of COG5512, is also predicted to be a nucleic-acid binding protein [14]. Binding to nucleic acid could be relevant to the role for DciA in DNA replication given the numerous protein-nucleic acid complexes that form during this process. We tested the DNA binding activity of purified DciA_Mtb_ protein (S7A Fig) in electromobility shift assays (EMSAs) with ^32^P-radiolabeled DNA. Due to its role in DNA replication (Figs 3 and 5), we first tested whether DciA_Mtb_ was able to bind *oriC* DNA *in vitro*. DciA_Mtb_ was able to bind and shift a 553 basepair (bp) dsDNA fragment containing *Mtb oriC* DNA [33] (Fig 6A). Given its high isoelectric point and the negative charge of DNA, we hypothesized that DciA_Mtb_ would be able to bind any DNA sequence and not just *oriC*. Indeed, we found that DciA_Mtb_ was also able to shift a 333 bp dsDNA sequence from a site in the genome distantly located from *oriC* (S7B Fig). DciA_Mtb_’s DNA binding activity is not limited to dsDNA sequences as DciA_Mtb_ was also able to bind and shift a 72 nucleotide single-stranded DNA (ssDNA) oligo (S7C Fig). These data indicate that DciA_Mtb_ can bind diverse double and single stranded DNA molecules *in vitro*, including *oriC*. This sequence independent DNA-binding activity could relate to the role of DciA_Mtb_ in DNA replication.

### DciA_Mtb_ associates with proteins involved in DNA replication and repair

We next identified the mycobacterial proteins that associate with DciA_Mtb_ by performing co-immunoprecipitation mass spectrometry (co-IP/MS). We engineered a strain of *M*. *smegmatis* that encodes an HA-tagged version of *dciA*_*Mtb*_ (HA-DciA_Mtb_) as its only *dciA* allele (S1 Table). We generated cell lysate from this strain and immunoprecipitated HA-DciA_Mtb_ along with associated protein complexes using an anti-HA antibody conjugated agarose (Sigma). After eluting with HA peptide, eluates were separated by SDS-PAGE, silver-stained, and bands specific to the HA-DciA_Mtb_ lane (S7D Fig) were isolated and analyzed by MS. We observed a similar banding pattern when we performed these experiments with DNAse-treated cell lysates (S7E Fig). The most abundant band on the silver-stained gel contained ClpX (S7D Fig), a component of the essential ClpXP protease. The association of DciA_Mtb_ with Clp protease may explain our inability to detect native DciA proteins by western blot. In addition to components of the Clp protease, we also found that DciA_Mtb_ associates with a number of proteins involved in DNA replication and repair (Fig 6B, S4 Table).

### DciA_Mtb_ interacts directly with DnaB

Since we have shown that DciA_Mtb_ is involved in DNA replication (Fig 5), we sought to confirm the association of DciA_Mtb_ with the co-immunoprecipitated DNA replication proteins. These proteins included gyrase, DnaX (τ clamp-loader subunit of DNA Pol III), DnaB (replicative helicase), and DnaA (replication initiator protein). To prioritize our studies, we identified DNA replication proteins that are conserved in *E*. *coli* and *B*. *subtilis* but do not yet have known homologs in mycobacteria, namely DnaA regulators and DnaB helicase loaders (Table 1). Since DnaA and DnaB were both found to associate with DciA_Mtb_ through co-IP/MS, we first tested whether DciA_Mtb_ directly interacts with these proteins.

**Table 1:** Review of *E*. *coli* and *B*. *subtilis* DNA replication proteins that are conserved in *Mtb*.

Role of Protein	*E*. *coli*	*B*. *subtilis*	Present in *Mtb*?	Mycobacterial publications
Chromosomal replication initiator protein	DnaA	DnaA	Yes	[25,33]
Replicative DNA helicase	DnaB	DnaC	Yes	[34–36]
Helicase Loaders	DnaC	DnaI*	No	
Primosomal proteins	DnaT*	DnaB*	No	
		DnaD*		
DNA Polymerase I	PolI	PolI	Yes	[37]
DNA Polymerase III a subunit (polymerase activity)	PolIIIα	PolC	DnaE1	[8,38]
		DnaE	DnaE2	[5,39]
Primase, primer synthesis	DnaG	DnaG	Yes	[40]
DNA Polymerase III β processivity clamp	DnaN	DnaN	Yes	[15,38,41]
DNA Polymerase III ε subunit, 3’ to 5’ proofreading	DnaQ	Encoded in PolC	Yes	[8,38]
Clamp loaders, τ and γ subunits of DNA Polymerase III holoenzyme	DnaX	DnaX	DnaZX	[38,41]
Regulators of DnaA activity	Hda	YabA	No	
	SeqA	SirA	No	
	DiaA	SoJ	No	
Replication Restart	PriA	PriA	Yes	[17]
	PriB		No	
	PriC			
Chromosomal Partitioning	ParA	Soj	Yes	[42–44] and others [42,45]
	ParB	Spo0J	Yes	
	Smc	Smc	Yes	
Single stranded binding protein	SSB	SSB	Yes	[46,47]
General DNA binding proteins	HU	HBsu	HupB,	[30–32,48–50]
	H-NS		H-NS	
	IHF		IHF	
			Lsr2	
Terminator protein	Tus	RTP	No	

The naming of proteins in *Mtb* follows *E. coli* nomenclature unless indicated

* also involved in replication restart

We performed pull-down experiments similar to the co-IP approach described earlier, but using purified recombinant HA-DciA_Mtb_, DnaB, and DnaA. We immobilized HA-DciA_Mtb_ onto anti-HA agarose and added DnaA or DnaB. Analysis of the protein complexes eluted with HA peptide showed that HA-DciA_Mtb_ pulls down both DnaA (Fig 6C lane 4) and DnaB (Fig 6C lane 6), but not a negative control protein, Rel_Mtb_^1-394^ (S7F Fig). Since HA-DciA_Mtb_, DnaA, and DnaB can all bind DNA, we tested whether the interactions between these proteins were dependent on nucleic acid by performing the same pull-down experiments using recombinant proteins purified from Benzonase-treated lysates to degrade nucleic acids. When proteins purified from Benzonase-treated lysates were used, HA-DciA_Mtb_ failed to pull down DnaA (Fig 6C lane 5) but retained its interaction with DnaB (Fig 6C lane 7). Therefore, DciA_Mtb_ interacts directly with DnaB but depends on nucleic acid to associate with DnaA.

We confirmed that DciA_Mtb_ and DnaB directly interact by performing the reciprocal pull-down with purified FLAG-tagged DnaB (DnaB-FLAG) immobilized as bait and DciA_Mtb_ as prey (Fig 6D). We performed Bio-Layer Interferometry (BLI) to quantify the affinity of the interaction between DnaB and DciA_Mtb_. The association and dissociation of varying concentrations of DciA_Mtb_ to biotinylated DnaB loaded onto streptavidin-coated biosensor pins (ForteBio) were measured (Fig 6E) and an affinity constant (K_D_) of 210.9 ± 8.19 nM was calculated (fit R^2^ = .9018) (S7G Fig). These data demonstrate that DnaB and DciA_Mtb_ interact in a dose-dependent manner. The calculated affinity constant in the nanomolar range suggests that the interaction could occur under physiological conditions, although cellular concentrations of DnaB and DciA_Mtb_ need to be quantified to confirm this.

### DciA_Mtb_ can affect the interaction between DnaB and DnaA

In *E*. *coli*, the interaction between DnaA and DnaB is required for efficient loading of DnaB [51,52]. The result that DciA_Mtb_ directly binds DnaB and can indirectly associate with DnaA led us to probe whether DciA_Mtb_ affects DnaB-DnaA complex formation. *Mtb* DnaA has been shown to interact with the N-terminus of DnaB (residues 1–206) [36], and we confirmed that the full length *Mtb* DnaB-FLAG and DnaA-HA proteins directly interact in our system (Fig 6F lane 1 and Fig 6G lane 6). To determine the effect of DciA_Mtb_ on the DnaA-DnaB interaction, we performed pull-downs with DnaA-HA as bait and DnaB-FLAG as prey in the presence of varying amounts of DciA_Mtb_. All pull-downs were performed using proteins purified from Benzonase-treated lysates to exclude contributions from nucleic acid interactions. As increasing amounts of DciA_Mtb_ were added, DnaA-HA pulled down more DnaB-FLAG (Fig 6F and 6H). These results indicate that DciA_Mtb_ facilitates DnaA-DnaB complex formation. In contrast, when DnaB-FLAG was immobilized as bait and DnaA-HA was added as prey, increasing the amount of DciA_Mtb_ present did not change the amount of DnaB-FLAG that associated with DnaA-HA (Fig 6G and 6H). Therefore, DciA_Mtb_ can affect DnaB-DnaA complex formation, but this is dependent on which protein is immobilized. One possible explanation for this observation is that DnaB, which functions as a hexamer [34], is unable to properly hexamerize while immobilized as bait, affecting DciA_Mtb_ activity. The ability of DciA_Mtb_ to positively affect the DnaB-DnaA interaction suggests that DciA_Mtb_ promotes rather than inhibits DNA replication, which is consistent with the observations that *dciA*_*Mtb*_ depletion leads to decreased DNA synthesis and DNA content (Fig 5).

### DciA_Mtb_ is enriched at *oriC* in *M*. *smegmatis*

The ability of DciA_Mtb_ to affect the association of DnaB with DnaA suggests that DciA functions during DNA replication initiation. To test whether DciA is enriched at *oriC* where initiation occurs, we performed chromatin immunoprecipitation quantitative PCR (ChIP-qPCR) experiments. These experiments were performed with log-phase cultures of *M*. *smegmatis* strains that express HA-tagged DciA (HA-DciA), untagged DciA (no tag), and HA-tagged CarD (HA-CarD), a mycobacterial transcription factor that associates with RNA Polymerase and is known to localize to every promoter throughout the *M*. *smegmatis* genome [53]. Protein-nucleic acid complexes were immunoprecipitated from each culture using anti-HA resin and co-immunoprecipitated DNA was probed for sequences specific for *oriC* (S8A Fig), the *rplN* promoter, and internal to the *sigA* gene using qPCR. Enrichment of sequences within a given sample was determined relative to the no tag control.

As expected, only DNA fragments containing promoters (*oriC* and *rplN* promoter) were specifically and significantly enriched for following immunoprecipitation with HA-CarD (S8B Fig). The only DNA fragments that were significantly enriched for following immunoprecipitation of HA-DciA_Mtb_ were those containing the *oriC* (Fig 6I). As a control, no sequences were enriched in input samples before immunoprecipitation (Fig 6I and S8C Fig). The specific enrichment of DciA_Mtb_ at *oriC* and not at other areas of the chromosome indicates that DciA_Mtb_ is involved in DNA replication initiation, which is consistent with its role in affecting the interaction between DnaB and the DnaA initiator protein (Fig 6F–6H).

### The C-terminus of DciA_Mtb_ is similar to the DnaA N-terminal domain

**Fig 7:**
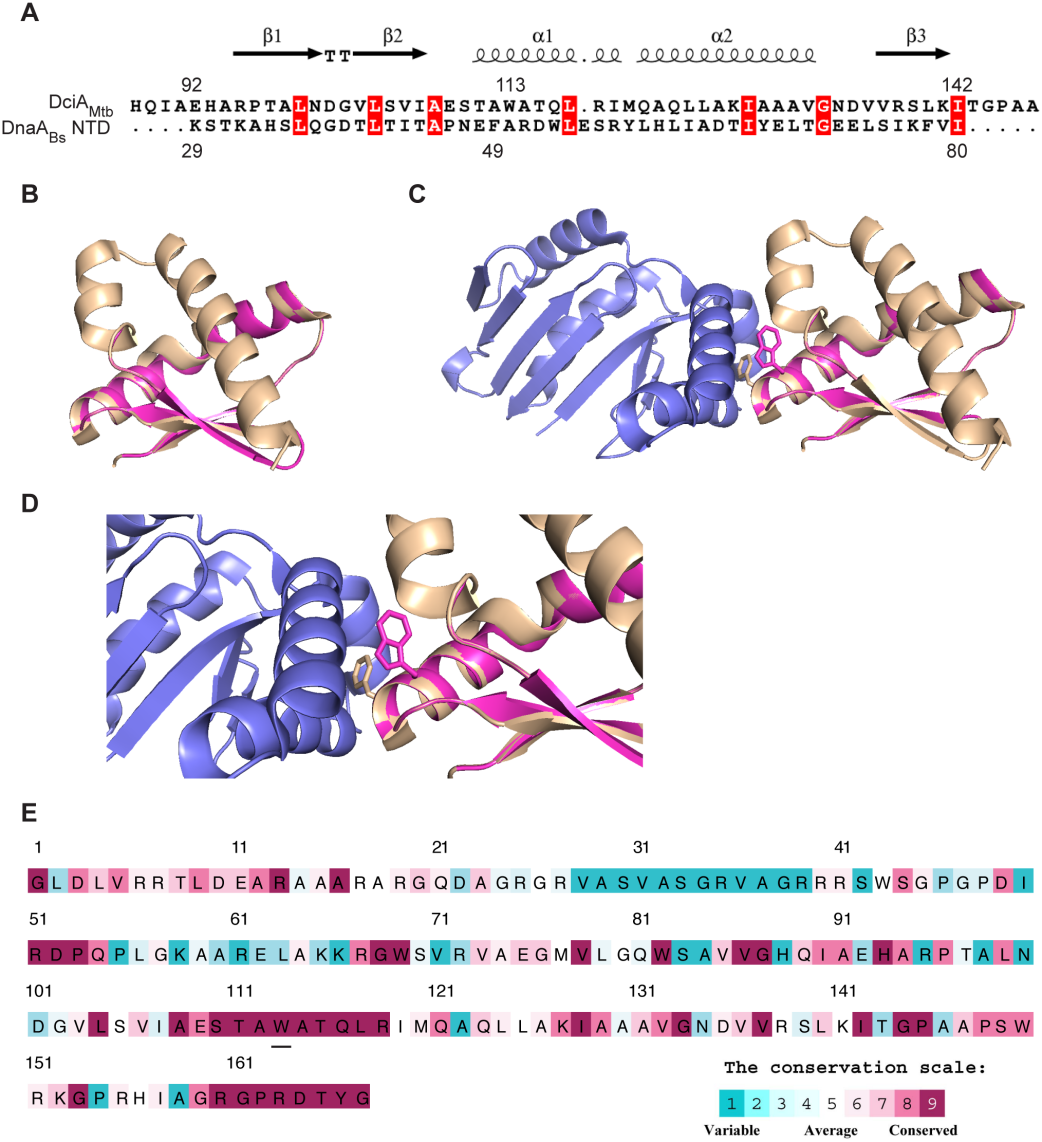
The C-terminus of DciA contains a structurally predicted DnaA NTD-Like (DANL) domain. (A) Alignment generated by Phyre2 of the DciA_Mtb_ DANL domain with *B*. *subtilis* DnaA NTD from PDB:4TPSD. Red boxes indicate identical residues, while structural predictions above the alignment refer to the predicted structure of DciA_Mtb_ (arrows for beta sheets, loops for alpha helices, and TT for the turn between the two beta sheets). (B) Structural model of DciA_Mtb_ DANL domain generated by Phyre2. DciA_Mtb_ DANL (residues 92–142, magenta) and the *B*. *subtilis* DnaA NTD (residues 29–80, gold, PDB:4TPSD) were aligned using PyMol. (C) The structural alignment is shown in the context of the SirA-DnaA complex (PDB:4TPS) with SirA in blue and the inter-facial locations of F49 of DnaA and W113 of DciA_Mtb_ highlighted. (D) Zoomed in image of (C). (E) Consurf analysis based on 150 non-redundant homologs of DciA_Mtb_ protein. Highly conserved residues are in magenta and variable residues are teal. The W113 residue is underlined. Due to a probable misannotation of the start codon, the beginning of actinobacterial DciA proteins share poor homology (Fig 1C). Therefore, Consurf analysis of DciA begins at the glycine that marks the beginning of homology across mycobacterial DciA proteins (at residue 21 in Fig 1C).

To investigate how DciA_Mtb_ facilitates the association between DnaA and DnaB, we used structural prediction tools to gain further insight into the protein architecture. Both Phyre2 [54] and I-TASSER [55] predicted that a region in the C-terminus of DciA_Mtb_ within DUF721 (~92–142 aa) is structurally similar to the N-terminal domain (NTD) of DnaA in *B*. *subtilis* (DnaA_*Bs*_, PDB: 4TPSD) and *E*. *coli* (DnaA_*Ec*_, PDB: 2E0GA) (Fig 7A and 7B). We named this region of DciA_Mtb_ the DnaA NTD-Like (DANL) domain. The DnaA NTD is responsible for many protein-protein interactions important for DNA replication, including the interaction of DnaA_*Ec*_ with DnaB, DiaA, and other DnaA_*Ec*_ monomers, the interaction of DnaA_*Bs*_ with SirA, and the interaction between *H*. *pylori* DnaA and HobA [51,52,56–58]. Phenylalanine 46 (F46) in DnaA_*Ec*_, which is equivalent to F49 in DnaA_*Bs*_, is specifically important for DnaA_*Ec*_ to load DnaB [57]. Though shifted by one residue, our structural alignment shows that a tryptophan at position 113 in DciA_Mtb_ (W113) is the closest aromatic amino acid to F49 in DnaA_Bs_ (Fig 7A, 7C and 7D). Consurf alignment [59] reveals that the region around W113 is one of two highly conserved regions of the DciA_Mtb_ protein that are both located in DciA_Mtb_’s C-terminus (Fig 7E). The position of W113 in the predicted structural model and the conservation of this region across DciA homologs suggest that W113 may be important for DciA_Mtb_ activity.

### A single point mutation in the DciA_Mtb_ DANL domain results in similar phenotypes as *dciA* depletion

**Fig 8:**
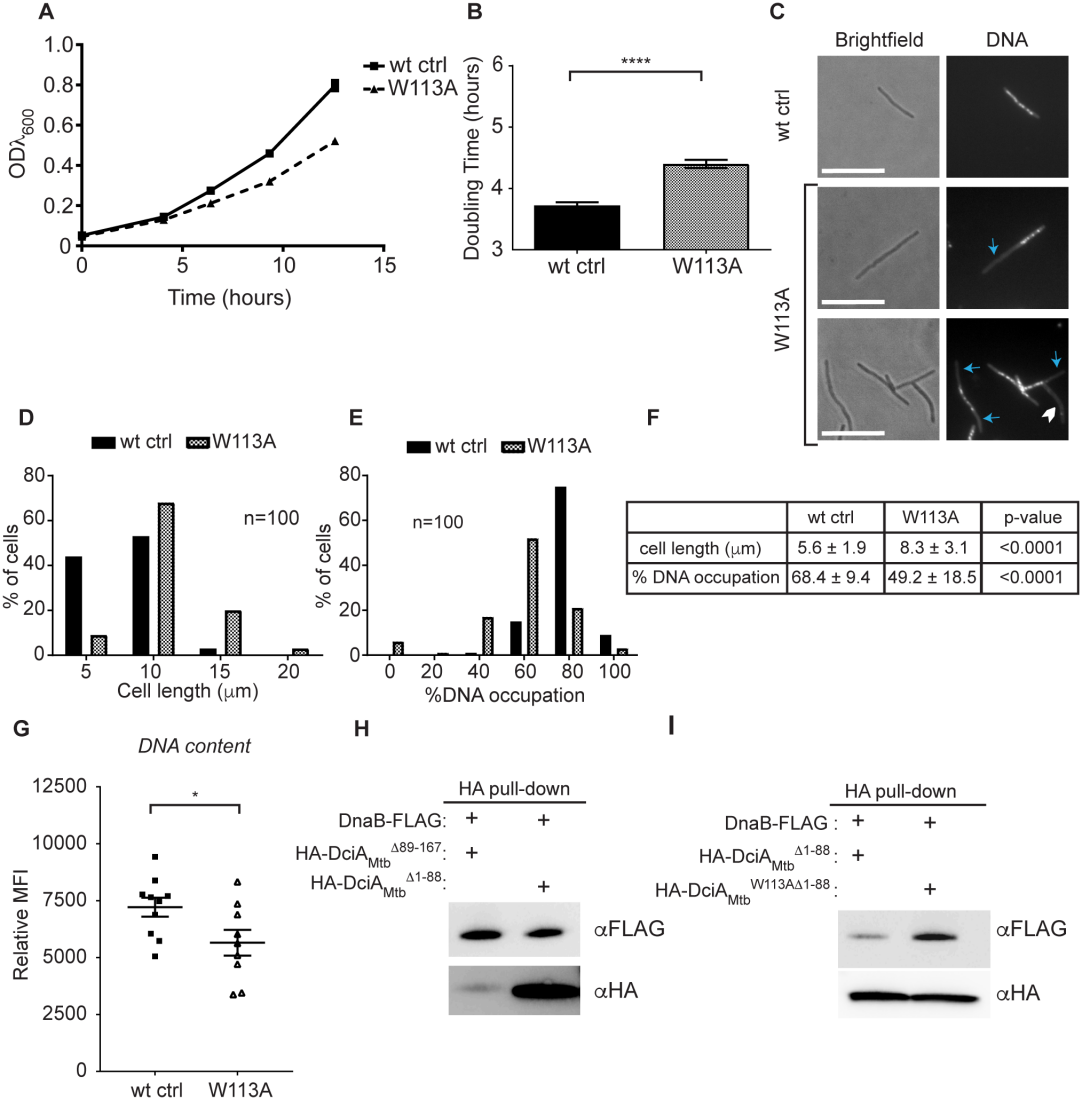
Mutating a conserved tryptophan in the DciA DANL domain causes similar phenotypes as *dciA*_*Mtb*_ depletion. (A) Representative growth curve of the *M*. *smegmatis* W113A strain (n = 3) and the wild-type control (wt ctrl, n = 4). Symbols represent means ± SEM. (B) Doubling times calculated from three independent experiments, bars indicate means ± SEM (wt ctrl n = 9; W113A n = 9, p <0.0001). (C) Fluorescence microscopy of wt ctrl (top row) or W113A (bottom two rows) stained with DAPI for DNA. Blue arrows indicate areas free of DNA staining and the white chevron indicates an anucleate cell. Scale bars are 10 μm. Cell lengths (D) and % DNA occupation (E) of wt ctrl or W113A cells. (F) Means ± SD from data in (D) and (E). (G) Geometric mean fluorescence intensity (MFI) of DAPI-stained wt ctrl or W113A relative to an unstained sample determined by flow cytometry. Representative flow cytometry histograms are found in S6B Fig. Symbols represent each replicate, center values and error bars represent mean ± SEM, * p <0.05, **** p <0.0001, statistical significance determined by Student’s unpaired *t*-test. (H,I) Western blot analyses of eluates from pull-downs with DnaB-FLAG as prey and either HA-DciA_Mtb_^Δ89–167^ (N-terminus) or HA-DciA_Mtb_^Δ1–88^ (C-terminus) (H) or HA-DciA_Mtb_^Δ1–88^ or HA-DciA_Mtb_^W113AΔ1–88^ (I) as bait. Each pull down was repeated at least three times with different preparations of purified proteins.

To test if W113 in the DciA_Mtb_ DANL domain is important for DciA_Mtb_ function, we engineered *M*. *smegmatis* to expresses a version of DciA_Mtb_ where the W113 is mutated to an alanine (W113A) as its only allele of *dciA*. The W113A mutation leads to a growth defect (Fig 8A and 8B), elongated cellular and abnormal nucleoid morphologies (Fig 8C–8F), and decreased DNA content (Fig 8G; S6B Fig). The observation that the mutation of a single residue can cause similar phenotypes to those observed during *dciA*_*Mtb*_ depletion confirms that the *dciA*_*Mtb*_-depletion phenotypes were not due solely to the depletion of an essential protein. These experiments also show that the W113 residue, which is located within the region of DciA_Mtb_ that is predicted to be structurally similar to the protein-protein interaction domain of DnaA, is important for DciA_Mtb_ activity.

### The W113A mutation affects the binding of the DciA_Mtb_ C-terminus to DnaB

In order to determine how W113 is contributing to DciA_Mtb_ function, we purified DciA_Mtb_^W113A^ protein and tested its ability to perform the functions we have assigned to DciA_Mtb_. DciA_Mtb_^W113A^ was able to bind and shift DNA (S9A Fig) at similar concentrations to DciA_Mtb_ wild-type protein (Fig 6A). DciA_Mtb_^W113A^ was also able to bind DnaB in the presence (S9B Fig) and absence (S9C Fig) of nucleic acid, as well as affect DnaA-DnaB complex formation similarly to wild-type DciA_Mtb_ (S9D–S9F Fig). The DNA and DnaB binding activities of DciA_Mtb_^W113A^ indicate that the DciA_Mtb_^W113A^ protein is functional, structurally intact, and not grossly misfolded.

Given the location of W113 in the predicted DANL protein-protein interaction domain, we were surprised that the W113A mutation did not affect the interaction between DciA_Mtb_ and DnaB. To test whether the DANL domain itself was involved in the interaction with DnaB, we purified the HA-tagged DciA_Mtb_ N-terminus (HA-DciA_Mtb_^Δ89–167^) and HA-tagged DciA_Mtb_ C-terminus (HA-DciA_Mtb_^Δ1–88^, containing the DANL domain) protein truncations, and tested their ability to bind DnaB. We found that both the N-terminus (HA-DciA_Mtb_^Δ89–167^) and the C-terminus (HA-DciA_Mtb_^Δ1–88^) of DciA_Mtb_ can individually bind DnaB-FLAG (Fig 8H). While we predicted the DANL-domain within the C-terminus of DciA_Mtb_ would bind DnaB based on its similarity to the DnaA NTD, we did not anticipate that the DciA_Mtb_ N-terminus would also interact with DnaB. This is particularly interesting since there are no known or predicted structures for the N-terminus of DciA_Mtb_. We were unable to isolate viable *M*. *smegmatis dciA*_*Mtb*_^*Δ89–167*^ or *dciA*_*Mtb*_^*Δ1–88*^ mutants using the gene-switching approach described earlier, indicating that both halves of the protein contribute to DciA_Mtb_’s essential cellular function. Therefore, the ability of both the N- and C-terminus of DciA to associate with DnaB is unlikely due to redundant roles for these two regions of the protein.

We tested whether the DnaB-binding activity provided by the N-terminus of DciA_Mtb_ was precluding our ability to assess the contribution of W113 to the interaction with DnaB. To investigate this, we purified the HA-tagged DciA_Mtb_^W113A^ C-terminus (HA-DciA_Mtb_^W113AΔ1–88^) and tested its ability to bind DnaB-FLAG. We found that HA-DciA_Mtb_^W113AΔ1–88^ associated with more DnaB-FLAG than the wild-type HA-DciA_Mtb_^Δ1–88^ (Fig 8I). Therefore, the W113A mutation in DciA_Mtb_ affects the association of the C-terminal DANL domain with DnaB and it is possible that the W113A *M*. *smegmatis* mutant strain could have defects due to altered DnaB binding by DciA_Mtb_^W113A^.

## Discussion

DciA proteins were recently discovered in an evolutionary and phylogenetic analysis that defined them by the presence of the DUF721/PF05258 domain [12]. Two classes of *dciA* genes were identified, those located in the *dnaN-recF* operon and those located elsewhere. Using *P*. *aeruginosa dciA* (*dciA*_*Pa*_) as an example of the second class of *dciA* genes, Brezellec *et al*. showed that DciA_Pa_ can associate with DnaB in a bacterial two-hybrid assay and that DNA replication is blocked during DciA_Pa_ depletion in *P*. *aeruginosa* [12]. However, the molecular details of how DciA associates with DnaB, the mechanisms by which DciA facilitates replication, whether DciA performs other activities in the cell, and whether the findings for DciA_Pa_ hold true for the other class of DciA proteins remained unknown.

In this study we address these gaps in knowledge beginning with our discovery that DciA_Mtb_, a member of the first class of DciA proteins, is an essential component of the DNA replication machinery that directly interacts with the DnaB helicase. Through detailed mechanistic work, we then expand on the work in *P*. *aeruginosa* to show that DciA localizes to the *oriC*, directly binds DNA, and affects the association of DnaB with DnaA, likely contributing to a role during DNA replication initiation. We also assign a role to DUF721 as a DnaA-NTD-like (DANL) protein-protein interaction domain and identify a tryptophan within the DANL domain that is critical for DciA_Mtb_ function *in vivo* and can affect DnaB binding *in vitro*. We find that both the N-terminus and C-terminus of DciA_Mtb_ are able to directly bind DnaB, indicating a complicated association between DciA and the replication machinery that will be the focus of future studies. The DANL domain of DciA_Mtb_ adds to the examples of DNA replication proteins across bacteria that share structural similarity despite divergent sequences (e.g. HobA and DiaA [58] as well as winged-helix domains of bacterial, mammalian, and archaeal DNA replication proteins [60,61]). It also remains possible that the DciA DANL domain interacts with additional proteins beyond DnaB, which will be explored in future studies.

To study the role of DciA in mycobacteria, we used a Tet-DciA strain that depletes *dciA*_*Mtb*_ transcripts following the removal of ATc from the media. *dciA*_*Mtb*_ transcripts were depleted by 16 hours following the removal of ATc, at which point we observed abnormal nucleoid morphology. The abnormal nucleoid morphology is the earliest phenotype observed during *dciA*_*Mtb*_ depletion and is followed by elongated cell lengths at 24 hours and then slower growth between 24 and 36 hours of *dciA*_*Mtb*_ depletion (S2 Fig). These data suggest that the defect in DNA replication that leads to abnormal nucleoid morphology results in the cell cycle block and a subsequent growth defect. Live-cell imaging revealed that both shorter and longer *dciA*_*Mtb*_-depleted cells underwent division, leading to increased heterogeneity among cell birth lengths and indicating a dysregulation of cell size and division (S1 and S2 Movies, S3 Fig). It is unknown what mechanisms control cell size in mycobacteria but our data implicates DciA in affecting the coordination of cell division and cell size. Since this is the first time a mycobacterial DNA replication mutant has been analyzed by live-cell imaging, future studies will determine if this dysregulation of cell size control is a general feature of mycobacterial DNA replication mutants or if it is DciA-specific. These studies will shed light onto the mechanisms that control mycobacterial cell size and cell cycle.

Helicase loaders have not been identified in the majority of bacterial phyla, making it tempting to assign this role to DciA. However, DciA homologs do not encode ATPase domains and, thus, do not fit the definition of a helicase loader [2]. Their small size, association with the replicative helicase, and importance in DNA replication are more reminiscent of the primosomal proteins *B*. *subtilis* DnaD and *E*. *coli* DnaT [18,62]. However, DnaD is present in the cell at very high levels [18], and neither DnaD nor DnaT has a high isoelectric point or a structurally predicted DANL domain. Therefore, DciA is unique from any protein that has been shown to facilitate DnaB association with replication complexes. There are two possible explanations for the lack of identified helicase loaders in mycobacteria. DnaB could be loaded by DciA_Mtb_ through a novel mechanism that is independent of any external ATPase activity and thereby replaces the need for a canonical helicase loader. Alternatively, DciA_Mtb_ could function in concert with an unknown ATPase in order to load the helicase. Our co-IP experiments identified a few known and hypothetical ATPase and ATP-binding domain containing proteins (S4 Table). Future studies will investigate the interactions of DciA_Mtb_ with these proteins and their roles in replication, as well as whether DciA_Mtb_ affects activities of DnaB outside of its interaction with DnaA (Fig 6F and 6H).

Our studies also revealed that DciA_Mtb_ can interact with single and double-stranded DNA *in vitro*. DciA homologs have high isoelectric points that likely mediate DNA-binding (Fig 6A). Integration host factor (IHF) also has a high isoelectric point and has been shown in *E*. *coli* to bind and bend *oriC* DNA [63], possibly to bring DnaA-boxes closer together and promote replication initiation [64]. The effect of IHF on *oriC* in mycobacteria has not yet been studied, but Lsr2, which has a high isoelectric point and is the mycobacterial functional analog of H-NS, is able to bind to open reading frames proximal to *oriC* [50]. ChIP-qPCR experiments showed that despite the ability of DciA_Mtb_ to bind DNA without sequence specificity, it is enriched at the *oriC*. This suggests that the specific localization of DciA_Mtb_ to *oriC* relies on its association with DnaB and the DNA replication initiation complex.

We originally identified DciA_Msm_ as being upregulated in response to DNA damage [13]. Although we do not know the exact role for DciA in the response to DNA damage, one can imagine a number of possibilities. First, DNA damage and DNA replication are inextricably linked, as DNA damage can disrupt the movement of the replisome and the repair of DNA damage involves proteins that are also involved in DNA replication. Second, DciA_Mtb_ could have a role in replication restart, which involves the loading of the replicative helicase onto non-*oriC* DNA and is dependent on proteins that bind DNA in a structure-dependent but sequence-independent manner in other bacteria [65]. Replication restart has not yet been studied in mycobacteria (Table 1). Lastly, DciA_Mtb_ could have a direct role in the repair of DNA damage independent of its role in DNA replication by interacting with DNA damage repair helicases. DciA_Mtb_ co-immunoprecipitated with a number of helicases involved in DNA repair (Fig 6B), and future studies determining whether DciA_Mtb_ directly interacts with these helicases will help to elucidate the connection between DciA and DNA damage responses.

Although we can detect immunoprecipitated HA-DciA_Mtb_ by MS, we have been unable to detect DciA_Msm_, DciA_Mtb_, or HA-DciA_Mtb_ from cell lysate by western blot using a polyclonal antibody that we raised against DciA_Mtb_ or an anti-HA antibody. Since ClpX, as well as ClpP1 and ClpC1, co-immunoprecipitated with HA-DciA_Mtb_ (S4 Table), one explanation for the low level of DciA_Mtb_ protein in the cell is that DciA_Mtb_ is a target of Clp protease, which is essential in mycobacteria [66]. ClpX and ClpC1 are adaptors for ClpP, which consists of ClpP1 and ClpP2 in mycobacteria. Raju *et al*. used *clpP1P2* depletion strains in *M*. *smegmatis* and *Mtb* to identify proteins that were present at higher levels during *clpP1P2* depletion, suggesting that they are targets of Clp [66]. DciA_Mtb_ protein levels were 2.53 fold higher (P value of .00285) during *clpP1P2* depletion in *Mtb*, further supporting that DciA_Mtb_ is targeted by Clp. Interestingly, DnaB was also found to be significantly enriched during *clpP1P2* depletion in *M*. *smegmatis* and *Mtb*. The ClpP protease is known to regulate cell cycle progression proteins in *Caulobacter crescentus*, where, like mycobacteria, ClpP is essential [67]. Therefore, it is possible that regulation of DNA replication proteins comprises one of the essential roles for Clp in mycobacteria.

Together, this study has elucidated functions of DciA_Mtb_ that are likely applicable to other DciA homologs. *dciA* genes are not found in *E*. *coli* and *B*. *subtilis*, the organisms that have traditionally been used to study bacterial replication, and as a result have remained undiscovered until recently, despite being widely conserved throughout the bacterial kingdom. Therefore, this study contributes to a developing new paradigm of bacterial DNA replication.

## Methods

### Bacterial strains and growth conditions

*Mtb* Erdman strain was grown at 37°C in Middlebrook 7H9 supplemented with 0.5% glycerol, 0.05% Tween 80, and 10% oleic acid/albumin/dextrose/catalase. *M*. *smegmatis* mc^2^155 and its derivatives were grown on LB agar plates supplemented with 0.5% dextrose and 0.5% glycerol and in LB broth supplemented with 0.5% dextrose, 0.5% glycerol, and 0.05% Tween 80 except in the [5,6-^3^H] uracil experiment, in which cells were grown in Middlebrook 7H9 supplemented with 0.5% dextrose, 0.5% glycerol, and 0.05% Tween 80 and for live-cell imaging, in which *M*. *smegmatis* was grown in Middlebrook 7H9 supplemented with 0.05% Tween 80, 10% ADC (albumin, dextrose, catalase), and 0.2% glycerol. All bacterial strains, plasmids, and primers used in this study are described in detail in S1–S3 Tables.

#### Antibiotics and chemicals

50 μg/ml Hygromycin (Invitrogen), 20 μg/ml Kanamycin (GoldBio), 12.5 μg/ml zeocin (Invitrogen), 0.2% acetamide (Sigma), and 50 ng/ml of anhydrous tetracycline (ATc) (Sigma) were used unless otherwise indicated. H_2_O_2_, MMS, streptomycin, isoniazid, rifampicin, ciprofloxacin (all from Sigma) were used at the indicated concentrations.

### Quantitative real-time PCR (qRT-PCR)

RNA was extracted from mycobacteria using Trizol (Invitrogen) followed by high salt and isopropanol precipitation. Contaminating genomic DNA was removed using the TURBO DNA-free kit (ThermoFisher Scientific), cDNA was synthesized using Superscript III (Invitrogen), and iTaq Universal SYBR Green Supermix (Bio-Rad) was used in qRT-PCR reactions. Primers used to amplify *16S* rRNA from *Mtb* and *M*. *smegmatis*, *dciaA*_*Mtb*_ *(Rv0004)*, *dciA*_*Msm*_ *(MSMEG_0004)*, and *M*. *smegmatis dnaA*, *dnaN*, *MSMEG_0002*, *recF*, *gyrB* and *sigA* are found in S3 Table. Levels of *dciA*_*Msm*_, *dciaA*_*Mtb*_, *dnaA*, *dnaN*, *MSMEG_0002*, *recF*, and *gyrB*, were normalized to either *16S rrnA* or *sigA* transcript levels as previously described [68].

### Fluorescence microscopy

*M*. *smegmatis* was collected, washed once with equal volume Phosphate Buffered Saline (PBS), resuspended in equal volume 1 μg/ml FM1-43FX (Thermo Fisher) diluted in PBS and incubated for 20 minutes at 37°C shaking. Cells were then fixed with 3% paraformaldehyde in PBS for 30 minutes shaking at 37°C. Fixed cells were applied to 0.1% poly-L-lysine (Sigma) treated multitest slides (MP Biomedicals) and then washed once with PBS. Cells were permeabilized by treatment with 2 mg/ml lysozyme (Sigma) at 37°C for 30 minutes followed by 0.1% Triton-X 100 (Sigma) for exactly five minutes at room temperature [69]. Cells were rinsed with PBS and stained with DAPI (Thermo Fisher) diluted to 5μg/ml with Slow Fade Antifade Equilibration Buffer (ThermoFisher) and mounted using the Slow Fade Antifade Kit according to the manufacturer’s instructions. Slides were visualized with a Zeiss Axioskop 2 Mot Plus equipped with an Axiocam MRm monochrome camera and a 100X, 1.4 numerical aperature Zeiss Plan Apochromat oil objective and images were acquired using Axiovision 4.6 software, or using an upright Zeiss Axio Imager M2 fluorescence microscope and the Zen Blue image acquisition software.

### Live-cell imaging

*M*. *smegmatis* cells were grown to log phase overnight with shaking at 37°C. *M*. *smegmatis* cells were filtered through a 10 μm filter to remove clumps before being loaded into a custom polydimethylsiloxane (PDMS) microfluidic device, as before [26]. The viewing device incorporates a main microfluidic channel for continuous flow for growth media, with a height of approximately 10–17 μm, and viewing chambers with a diameter of 60 μm and a height of 0.8−0.9 μm. 2% DMSO and 0.0625 mg/ml FM4-64 are present in the flowed medium, to stain septal membranes.

The microfluidics device was placed on an automated microscope stage housed within an environmental chamber maintained at 37°C. *M*. *smegmatis* cells were imaged for up to 40 hours using a widefield DeltaVision PersonalDV (Applied Precision, Inc) with a hardware-based autofocus. Cells were illuminated with an InsightSSI Solid State Illumination system every 15 minutes: FM4-64 was visualized with 475nm excitation and 679 nm emission wavelengths, and cells were imaged using transmitted light brightfield imaging. Each set of images was illuminated with identical imaging conditions that were optimized to decrease phototoxicity inherent in long-term fluorescent imaging of live cells.

Tet-DciA was grown in the presence of hygromycin and ATc overnight and for the duration of the DciA_Mtb_ replete control movies (S1 Movie). Tet-DciA was grown in the presence of hygromycin and ATc overnight followed by growth in the presence of hygromycin alone for 10.5 hours prior to imaging and for the duration of imaging (S2 Movie). For analysis, all Tet-DciA cells grown in depleting conditions were born at least 24 hours after ATc was removed from the media.

### Transmission electron microscopy with thin-sectioning

*M*. *smegmatis* was fixed in 2% paraformaldehyde/2.5% glutaraldehyde (Polysciences Inc.) in 100 mM sodium cocadylate buffer, pH 7.2 for 1 hour at room temperature. Samples were washed in sodium cacodylate buffer and postfixed in 1% osmium tetroxide (Polysciences Inc.) for 1 hr. Samples were then rinsed extensively in dH_2_0 prior to en bloc staining with 1% aqueous uranyl acetate (Ted Pella Inc.) for 1 hr. Following several rinses in dH_2_0, samples were dehydrated in a graded series of ethanol and embedded in Eponate 12 resin (Ted Pella Inc.). Sections of 95 nm were cut with a Leica Ultracut UCT ultramicrotome (Leica Microsystems Inc.), stained with uranyl acetate and lead citrate, and viewed on a JEOL 1200 EX transmission electron microscope (JEOL USA Inc.) equipped with an AMT 8 megapixel digital camera and AMT Image Capture Engine V602 software (Advanced Microscopy Techniques).

### [5,6-^3^H]-thymidine incorporation assays

Assays were carried out similarly to published reports [25,28], with a few modifications. *M*. *smegmatis* cells were collected at ODλ_600_ = 0.3, washed once with 7H9 media, and inoculated into fresh 7H9 media containing 1μCi/ml [5,6-^3^H]-uracil (Perkin Elmer). [5,6-^3^H]-uracil is converted to [5,6-^3^H]-thymidine and incorporated into DNA in mycobacteria [25,28].

After 20 and 60 minutes of labeling, 3 ml aliquots of cells were taken for processing to measure counts per minute (cpm) of ^3^H incorporated into DNA or for enumeration of bacterial counts by colony forming units (cfu). A 7H9 media plus [5,6-^3^H]-uracil sample was also collected. Aliquots for ^3^H cpm were treated with 0.3M KOH to hydrolyze RNA and incubated at 37°C for 24 hours. Macromolecules were then precipitated with ice cold 10% trichloroacetic acid (Sigma) and filtered onto glass 25 mm GF/C filters (GE Healthcare). Filters were dried under a heat lamp and submerged in 15 ml of Ultima Gold liquid scintillation fluid (Perkin Elmer). ^3^H counts per minute (cpm) were measured using a Beckman LS6000IC scintillation counter that was programmed to read each sample for 5 minutes. All presented data indicates the cpm of that sample minus the cpm from the processed media + [5,6-^3^H]-uracil control to subtract background, relative to the cfu of that sample. The efficiency of RNA hydrolysis by KOH treatment was confirmed by including a KOH-untreated control.

### Measuring DNA content by flow cytometry

Cells were fixed and permeabilized as described for fluorescent microscopy [69] with an additional RNaseI_f_ treatment (NEB), and stained with 100 μM DAPI diluted in water for 15 minutes at room temperature. Cells were then resuspended in PBS, sonicated, and passed through a 30μm filter to remove clumps before flow cytometry analysis. Samples were analyzed using a FACSAria (Becton Dickinson) and data were processed with FlowJo (Treestar). All cells (events) were included in the analysis of DAPI intensity.

### Protein purification

*Mtb dciA*_*Mtb*_, *HA-dciA*_*Mtb*_, *dciA*_*Mtb*_^*W113A*^, *HA-dciA*_*Mtb*_^*W113A*^, truncations of HA-*dciA*_*Mtb*_ and HA-*dciA*_*Mtb*_^*W113A*^, *dnaA*, and *HA-dnaA* were cloned into pGEX-6P (GE Healthcare Life Sciences, S2 and S3 Tables). The plasmids were transformed into BL21(DE3) (Novagen). *Mtb DnaB* and *DnaB-FLAG* were amplified from *Mtb* genomic DNA following the published cloning scheme[35] to exclude the native intein, cloned into pET SUMO (Invitrogen) and transformed into BL21(DE3) cells (Novagen). Rel_Mtb_^1-394^ was cloned into pET SUMO [70]. Transformed cells were grown to mid-logarithmic phase (ODλ_600_ 0.6–0.8) and induced with 1mM isopropyl β-D-1-thiogalactopyranoside (IPTG) (GoldBio) for 3 hours at 37°C. Cells were harvested by centrifugation and resuspended in lysis buffer supplemented with 1 mg/ml lysozyme and flash frozen. The lysis buffer for DnaB constructs was 50mM Tris-HCl pH8, 300mM NaCl, 5mM imidazole pH8 and 1mM β-mercaptoethanol (BME). The lysis buffer for DnaA was PBS, and for the DciA_Mtb_ constructs the lysis buffer was 0.25M Urea, 750mM NaCl, 2.7mM KCl, 10mM sodium phosphate dibasic heptahydrate, 2mM potassium phosphate supplemented with .01% Nonidet P-40 (Sigma) and .1% TritonX 100. After thaw, cells were sonicated, treated with 9 units per ml Benzonase (Sigma) and 6mM MgCl_2_ when indicated, and spun at 10,000g to clarify the lysate.

For the purification of DnaB and Rel_Mtb_^1-394^ lysate was incubated with Ni-NTA agarose (Qiagen), washed with 50mM Tris-HCl pH8, 300mM NaCl, 20mM imidazole pH8, and 1mM BME, and proteins were eluted with 50mM Tris-HCl, pH8, 300mM NaCl, 250mM imidazole pH8, and 1mM BME. The His-SUMO tags were cleaved with His-tagged Ulp1 enzyme. A second incubation with Ni-NTA agarose was used to bind His-Ulp1 and the cleaved His-SUMO tag, and un-tagged recombinant protein was collected as flow-through.

For the purification of DnaA and DciA_Mtb_ constructs, lysate was incubated with Protino Glutathione Agarose 4B (Macherey-Nagel), washed with wash buffer (PBS or DciA_Mtb_ lysis buffer plus .01% Nonidet P-40), and cleaved on beads with GST-tagged PreScission Protease (GE Healthcare) in 50mM Tris-HCl pH7, 150mM NaCl, 1mM EDTA, and 1mM DTT, with un-tagged recombinant protein collected as flow-through.

### Electromobility shift assays

DNA fragments (see S3 Table) containing *oriC*_*Mtb*_ [33] and *rrnAPL* [19] were amplified by PCR and products were gel-purified using QIAquick column (Qiagen). 250 ng of gel-purified dsDNA or 3x FLAG ssDNA oligo (see S3 Table) were labeled with T4 polynucleotide kinase (NEB) and [γ-^32^P]-ATP, and unincorporated [γ-^32^P]-ATP was removed using Illustra ProbeQuant G-50 microcolumns (GE Healthcare). 20,000 cpm of labeled probe, 10 μg BSA, and the indicated amounts of DciA_Mtb_ or DciA_Mtb_^W113A^ were mixed with buffer (50mM Tris-HCl pH7, 150mM NaCl, 1mM EDTA, 1mM DTT) in a total volume of 12μl and incubated for 20 minutes at room temperature. Samples underwent native electrophoresis in 4–20% nondenaturing TBE polyacrylamide gels (Invitrogen). The gels were dried and exposed to film for detection by autoradiography.

### Immunoprecipitation and pull-down experiments

For immunoprecipitation from *M*. *smegmatis* cell lysates, 1 liter cultures of mid-logarithmic phase *M*. *smegmatis* were washed with PBS and frozen at -80°C until processing. Cell pellets were resuspended in 10mls NP-40 Buffer (10 mM sodium phosphate, pH8, 150 mM NaCl, 1% Nonidet P-40, and Roche EDTA-free Complete protease inhibitor cocktail) and lysed using a Constant Systems Cell Disruptor (4 passes at 40k psi), and spun at 50,000g for 30 minutes to generate lysate. When indicated, some lysates were treated with DNase I (NEB) according to manufacturers instructions. 1 ml of lysate was added to 50 μL monoclonal anti-HA agarose (Sigma) and rotated at 4°C overnight. The anti-HA agarose was then washed 3 times with NP-40 buffer and immune complexes were eluted with 500 μg/ml HA peptide (Roche) in IP elution buffer (50 mM Tris-HCl, pH 7.5, 50 mM NaCl with Roche EDTA-free Complete protease inhibitor cocktail).

For pull-down experiments with purified protein, 0.3114 nmol of purified bait protein in 100 μL NP-40 buffer was bound to either 50 μL anti-HA agarose (Sigma) or 40 μL anti-FLAG M2 affinity gel (Sigma) and rotated for 5–6 hours 4°C. The bait-bound matrix was then washed 3 times with NP-40 buffer and twice molar excess (0.6228 nmol) of prey protein was added (unless otherwise indicated) in 275 μL NP-40 buffer and rotated at 4°C overnight. The matrix was then washed three times and immune complexes were eluted with either 500 μg/ml HA peptide or 150 μg/ml FLAG peptide in IP elution buffer as described above.

### Antibodies and western blot

For western blot analyses, FLAG-tagged proteins were detected with mouse monoclonal anti-FLAG clone M2 antibody (Sigma) and HA-tagged proteins were detected with mouse monoclonal anti-HA clone HA-7 (Sigma). To detect DciA_Mtb_, we used a rabbit polyclonal anti- DciA_Mtb_ antibody that was generated by Cocalico Biologicals, Inc. by raising rabbit anti-sera against purified DciA_Mtb_ protein. Western blots were visualized on a ChemiDoc Touch Imaging System (Biorad) as well as film and quantified using ImageLab software version 5.2.1 software (Biorad).

### Chromatin immunoprecipitation quantitative PCR (ChIP-qPCR)

50 mL cultures of *M*. *smegmatis* expressing either HA-tagged DciA_Mtb_ (HA-DciA_Mtb_), HA-tagged CarD (HA-CarD), or untagged DciA_Mtb_ (No tag) were grown to an ODλ_600_ of 0.6. Protein-nucleic acid complexes were crosslinked with 2% formaldehyde for 30 min at room temperature, crosslinking was quenched with 0.125M glycine, and cells were lysed in ChIP lysis buffer (50mM HEPES-KOH pH7.5, 140mM NaCl, 1mM EDTA, and 1% TritonX 100, 1X protease inhibitors (Roche), and 2mg/ml lysozyme). After sonication, 0.5 ml of lysate was kept as input fractions and the remaining lysate was rotated with anti-HA agarose (Sigma) overnight at 4°C. Anti-HA agarose was washed 2 times each with ChIP lysis buffer, ChIP lysis buffer plus 360mM additional NaCl, ChIP wash buffer (10mM Tris-HCl pH8, 250mM LiCl, 0.5% NP-40, 0.5% sodium deoxycholate, and 1mM EDTA), and TE buffer (10mM Tris-HCl pH8 and 1mM EDTA). All buffers were supplemented with 1X protease inhibitors. Protein-nucleic acid complexes were eluted from the anti-HA agarose twice with ChIP elution buffer (50mMTris-HCl pH8, 10mM EDTA, 1% sodium dodecyl sulfate, 1X protease inhibitors) for 10 min at 65°C with agitation. Inputs and eluates were incubated at 65°C overnight to reverse crosslinking. Two phenol-chloroform extractions were performed consecutively on inputs and eluates and DNA was precipitated with ethanol and resuspended in TE buffer. Inputs and eluates were diluted to 0.5896 ng/μl and 0.426 ng/μl, respectively. Quantitative PCR (qPCR) was performed with 1 μl of the diluted DNA using primers to amplify three DNA fragments in *oriC* (*oriC1*, *oriC2*, *oriC3*), a fragment in the promoter of *rplN*, and a fragment within the *sigA* coding region (*sigAIN*) (See S3 Table and S8A Fig).

### Biolayer interferometry

Recombinant DnaB and DciA_Mtb_ were purified from *E*. *coli* as described above. An additional dialysis step to remove Tris from the buffer was performed into 150mM NaCl, 10mM NaPO_4_ pH8, 1mM BME. DnaB was non-specifically biotinylated *in vitro* using EZ-Link NHS-PEG_4_-Biotin (ThermoFisher). Five molar excess of biotin relative to DnaB was used and incubated for 30 minutes at room temperature. Excess biotin was removed using Zeba Spin Desalting columns (ThermoFisher).

The Octet RED96 System was used to attain biolayer interferometry (BLI) progress curves. Assay buffer for all steps consisted of 150mM NaCl, 0.02% Tween-20, 0.1% bovine serum albumin, and 10mM NaPO_4_ pH8. Briefly, streptavidin (SA) biosensor pins (ForteBio) were first equilibrated by being dipped into assay buffer for a 180 second baseline step, and then captured 200nM biotinylated DnaB during a 200 second loading step, followed by a 180 second baseline step in assay buffer. After performing an additional 60 second baseline step in assay buffer, pins were dipped into DciA_Mtb_ protein samples for a 300 second association step, followed by a 300 second dissociation step in assay buffer. This series of 60 second baseline, 300 second association, and 300 second dissociation steps was performed for each concentration of DciA_Mtb_. Curves were corrected by subtracting double reference of both biotin-coated pins dipped into the DciA_Mtb_ wells and DnaB-coated pins dipped into buffer only. Data was analyzed on ForteBio Data Analysis 6.4. Processed data were fit globally for all concentrations of DciA_Mtb_ in a 1:1 kinetic binding model.

### Mass spectrometry

Bands generated by SDS-PAGE followed by staining with ProteoSilver Plus Silver Stain Kit (Sigma), were cut out and destained according to manufacturer’s instructions, and submitted to the Proteomics & Mass Spectrometry Facility at the Danforth Plant Science Center for trypsin digestion followed by LC-MS/MS analysis.

### Statistics

Prism6 (Graphpad Software, Inc.) was used to determine statistical significance of differences. Unpaired two-tailed Student’s *t*-test was used to compare two groups with similar variances. Unpaired two-tailed Student’s *t*-test with Welch’s correction was used to compare two groups with different variances. One-way analysis of variance (ANOVA) and Tukey’s multiple comparison test were used to determine significance when more than two groups were compared. When utilized, center values and error bars represent mean ± SEM. * p <0.05, ** p <0.01, *** p<0.001, **** p <0.0001.

## Supporting information

S1 Fig Transcript levels of genes encoded near the *oriC*. Fold change in transcript levels relative to 16S rRNA in *M*. *smegmatis* for *dnaA* (black bars), *dnaN* (orange bars), *MSMEG_0002* (blue bars), *recF* (grey bars), *dciA* (purple bars), and *gyrB* (white bars) from the same culture during log (log, average ODλ_600_ = 0.234) or stationary (stat., average ODλ_600_ = 2.092) phase where log phase levels were set to 1. Each bar represents mean ± SEM (n = 3). **** p <0.0001, ** p <0.01, * p <.05, ns is not significant. Statistical significance was determined by one-way ANOVA and Tukey’s multiple comparison test.
(TIF)

S2 Fig Cell length and % DNA occupation histograms during Tet-DciA continual log growth curves. Cell length and % DNA occupation histograms of Tet-DciA grown in the presence (black bars) or absence (grey bars) of ATc at the indicated time points during continual log growth curves. Accompanying tables display averages ± standard deviations from data depicted in histograms, along with p-values determined by Student’s *t*-test.
(TIF)

S3 Fig Analysis of live-cell imaging of Tet-DciA grown in replete and depleting conditions. (A,B) Box plots demonstrating the median length of 150 Tet-DciA cells grown in replete (+ATc) and 150 Tet-DciA cells grown in depleting (-ATc) conditions at birth (A) and division (B). (C) Table displays mean ± SD and (coefficient of variation) from data depicted in previous box plots and subsequent histograms, along with p-values comparing median lengths determined by Student’s *t*-test, **** p <0.0001. (D,E) Histograms of cell length at birth (D) or division (E) of the same 150 Tet-DciA cells grown +ATc (black bars) and -ATc (grey bars).
(TIF)

S4 Fig *dciA*_*Mtb*_ depletion phenocopies *dnaA* depletion in *M*. *smegmatis*. Growth of rgm36, an acetamide (acet.)-inducible *dnaA* depletion *M*. *smegmatis* strain (A) on plates and (B,C) in continual log growth curves with two biological replicates per condition per growth curve (total of n = 4 +acet., n = 4 –acet.). The mean ± SEM is graphed.
(TIF)

S5 Fig *dciA*_*Mtb*_ depletion does not phenocopy *ftsZ* depletion in *M*. *smegmatis*. Growth of csm362, a TetOn FtsZ depletion *M*. *smegmatis* strain (A) on plates and (B) in a continual log growth curve with four biological replicates per condition. The mean ± SEM is graphed.
(TIF)

S6 Fig DNA content as measured by DAPI staining and flow cytometry. (A) Representative flow cytometry histograms in the Pacific Blue channel, which measures DAPI fluorescence, of unstained Tet-DciA cells (black), DAPI stained Tet-DciA grown in depleted (-ATc, blue), replete (+ATc, red) conditions. Cells were collected at the 36 hour time point of a continual logarithmic growth curve. Histograms like this were used to calculate DAPI mean fluorescence intensity (MFI) plotted for several biological replicates shown in Fig 5D. (B) Representative flow cytometry histograms in the Pacific Blue channel of unstained cells (black), DAPI stained wild-type control (wt ctrl, orange), and W113A (purple) cells. Histograms like this were used to calculate DAPI MFI plotted for several biological replicates shown in Fig 8G.
(TIF)

S7 Fig Gel shift assays with diverse DNA substrates, SDS-PAGE of HA-DciA_Mtb_ co-immunoprecipitation eluate, and fits from biolayer interferometry. (A) GelCode Blue-stained SDS-PAGE gel of purified DciA_Mtb_ protein used in electromobility shift assays. Minor lower molecular weight protein bands were also identified as DciA_Mtb_ by mass spectrometry. (B,C) Autoradiographs of electromobility shift assay with DciA_Mtb_ protein binding 1.4ng of ^32^P-labeled 333 base pair *rrnAPL* double-stranded DNA (B) or 546pg of ^32^P-labeled 72 nucleotide oligo single-stranded DNA (C). All lanes contain ^32^P-labeled substrate DNA. Lane 1 contains no protein, while the amount of DciA_Mtb_ in the other lanes is indicated above each autoradiograph. (D,E) Silver-stained SDS-PAGE of proteins that co-immunoprecipitated with HA-DciA_Mtb_ in lysate from the *M*. *smegmatis* HA-DciA_Mtb_ or Δ*dciA*_*Msm*_ *attB*::tet*dciA*_*Mtb*_ (no tag) strains. (D) Arrow indicates band containing ClpX. (E) Lysates were treated with DNase I prior to immunoprecipitation. (F) GelCode Blue-stained SDS-PAGE of purified proteins that were used as input, proteins that came off in sequential washes (see Methods), and eluate from a pull-down experiment with HA-DciA_Mtb_ as bait and Rel_Mtb_^1-394^ as prey. Top arrow indicates size of Rel_Mtb_^1-394^, bottom arrow indicates size of HA-DciA_Mtb_. (G) The yellow, green, light blue, red, and dark blue curves are the same representative curves shown in Fig 6E of the association and dissociation of DciA_Mtb_ at the indicated concentrations to and from biotinylated-DnaB as measured by biolayer interferometry. The red lines are global fits for each corresponding concentration of DciA_Mtb_ calculated by ForteBio Data Analysis 6.4 software based on a 1:1 kinetic binding model.
(TIF)

S8 Fig *oriC* is not enriched in chromatin immunoprecipitation (ChIP) input samples and CarD is enriched at *oriC* and the *rplN* promoter, but not in the *sigA* coding region. (A) Schematic of DNA fragments generated by PCR using *ori1*, *ori2*, and *ori3* primers. (B) Fold enrichment in DNA fragments that co-immunoprecipitated with HA-CarD (αHA-IP) or are present in input samples (input) relative to the No tag strain. Bars represent mean ± SEM (n = 5 except No tag *sigAIN*, which is n = 4). (C) Fold enrichment in levels of DNA fragments containing oriC (*oriC1-3*) in input (non-immunoprecipitated) samples of lysates from strains expressing untagged DciA_Mtb_ (No tag, solid black bars), HA-DciA_Mtb_ (grey bars), and HA-CarD (white bars) strains. Data is represented as fold enrichment relative to No tag levels and shows that none of these DNA fragments were enriched prior to immunoprecipitation. **** p <0.0001, ns is not significant, statistical significance was determined by one-way ANOVA and Tukey’s multiple comparison test.
(TIF)

S9 Fig The W113A mutation does not abrogate molecular interactions with DciA_Mtb_. (A) Autoradiograph of EMSA with DciA_Mtb_^W113A^ protein and 4.8 ng *oriC*_*Mtb*_ dsDNA separated by native PAGE. All lanes contain ^32^P-labeled *oriC*_*Mtb*_ DNA. Amount of DciA_Mtb_^W113A^ in each lane is indicated. (B) GelCode Blue-stained SDS-PAGE of purified proteins that were used for inputs and eluates from pull-down experiments with DciA_Mtb_^W113A^ as bait and the indicated protein as prey. These proteins were not treated with Benzonase during purification. Top arrow indicates approximate sizes of DnaB and Rel_Mtb_^1-394^, bottom arrow indicates size of DciA_Mtb_^W113A^. (C) Western blot analysis of pull-downs with either HA- DciA_Mtb_ or HA- DciA_Mtb_^W113A^ as bait and DnaB-FLAG as prey. Proteins were treated with Benzonase during purification (D,E) Representative western blot of pull-downs with (D) DnaA-HA as bait and DnaB-FLAG as prey or (E) DnaB-FLAG as bait and DnaA-HA as prey and either no (lanes 1,6), 0.5x (lanes 2,7), 1x (lanes 3,8), 2x (lanes 4,9), or 4x (lanes 5,10) molar ratio of DciA_Mtb_^W113A^ relative to the bait. (F) Quantification of the ratio of prey-to-bait for triplicate western blots like those shown in (D) and (E) where the ratio for lanes with no DciA_Mtb_^W113A^ is set to 1 and the ratio for all other samples is relative to the lane with no DciA_Mtb_^W113A^. Symbols represent each replicate, center values and error bars represent mean ± SEM.
(TIF)

S1 Table List of bacterial strains used in this study. (DOCX)

S2 Table List of plasmids used in this study. (DOCX)

S3 Table List of primers used in this study. (DOCX)

S4 Table Proteins detected by mass spectrometry of bands cut from SDS-PAGE of HA-DciA_Mtb_ co-immunoprecipitation experiment depicted in S7 Fig. (XLSX)

S1 Movie Time-lapse microscopy of Tet-DciA grown in the presence of ATc and FM4-64 membrane stain. Cells were grown in a microfluidic device and images were acquired at 15 minute intervals. This movie tracks the growth and division of Tet-DciA grown in replete conditions.
(MOV)

S2 Movie Time-lapse microscopy of Tet-DciA grown in the absence of ATc for 10.5 hours prior to imaging. During imaging, cells were grown in the presence of FM4-64 membrane stain. Cells were grown in a microfluidic device and images were acquired at 15 minute intervals. This movie tracks the growth and division of Tet-DciA grown in *dciA*_*Mtb*_ depleting conditions.
(MOV)
