# Supplementary figures and images for: Rv0004 is a new essential member of the mycobacterial DNA replication machinery
Source: PLoS Genet. 2017 Nov 27;13(11):e1007115. doi: 10.1371/journal.pgen.1007115 (PMC5720831; doi:10.1371/journal.pgen.1007115)

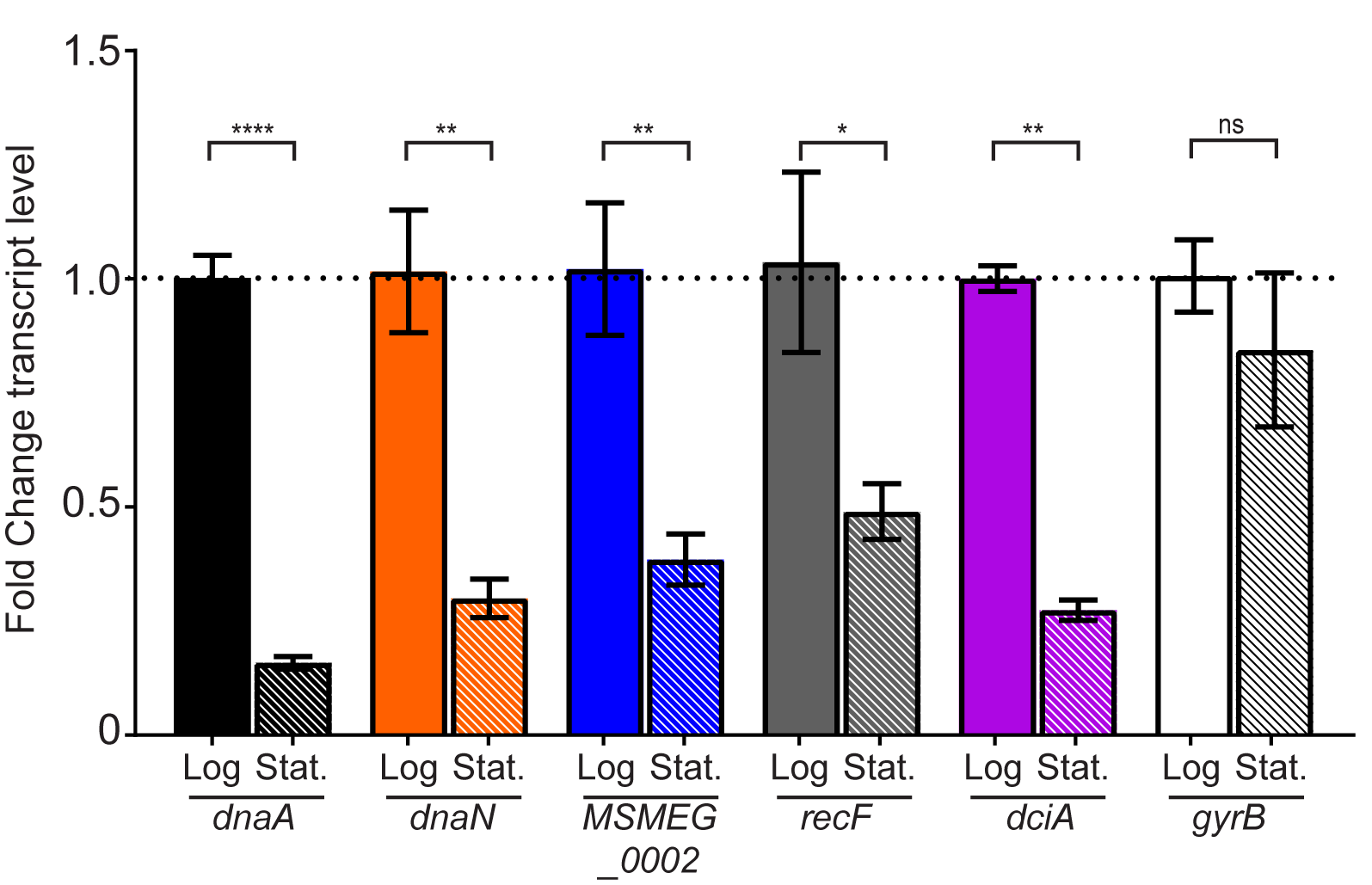

Supplement: S1 Fig — Fold change in transcript levels relative to 16S rRNA in M. smegmatis for dnaA (black bars), dnaN (orange bars), MSMEG_0002 (blue bars), recF (grey bars), dciA (purple bars), and gyrB (white bars) from the same culture during log (log, average ODλ600 = 0.234) or stationary (stat., average ODλ600 = 2.092) phase where log phase levels were set to 1. Each bar represents mean ± SEM (n = 3). **** p <0.0001, ** p <0.01, * p <.05, ns is not significant. Statistical significance was determined by one-way ANOVA and Tukey’s multiple comparison test. (TIF) [file pgen.1007115.s001.tif]

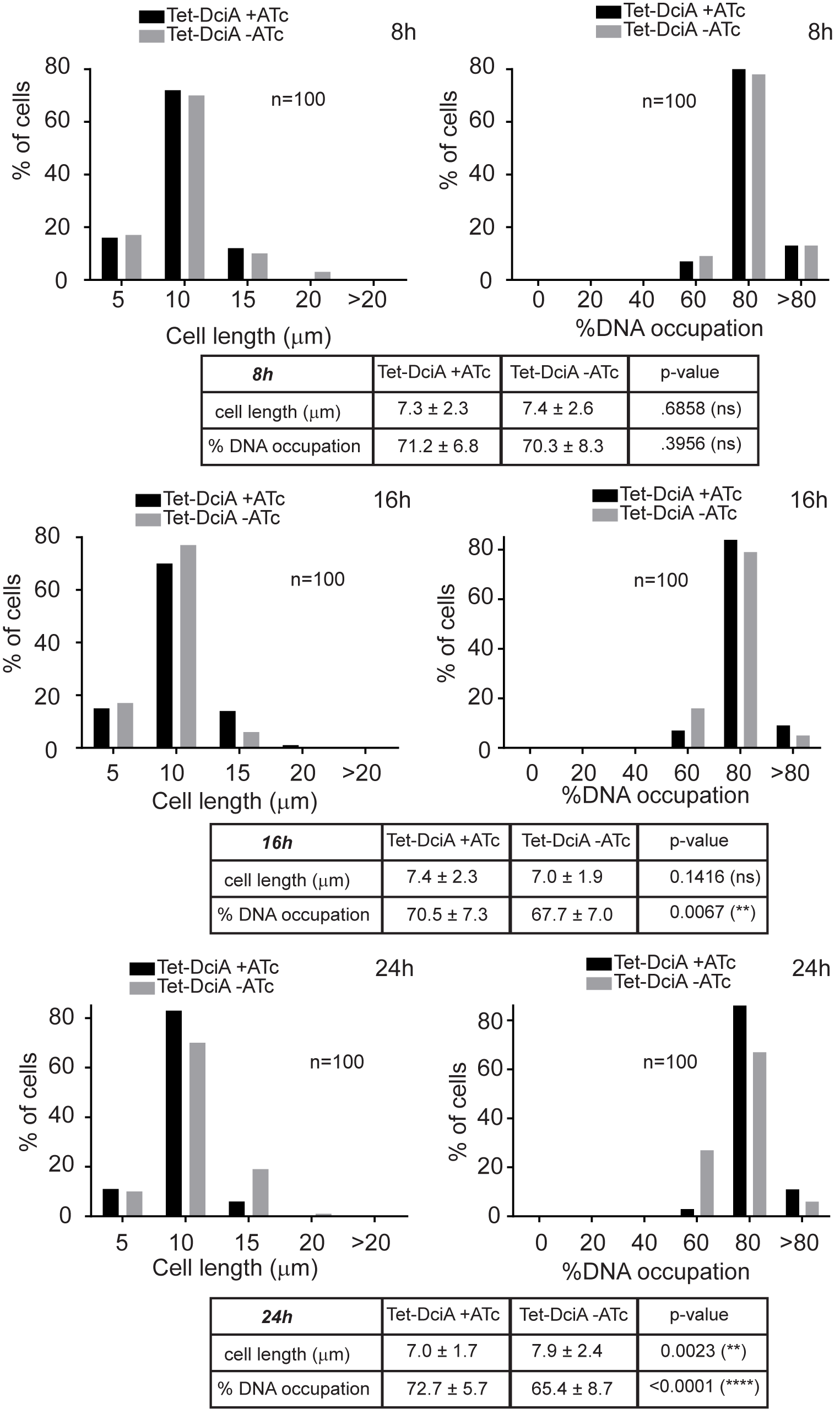

Supplement: S2 Fig — Cell length and % DNA occupation histograms of Tet-DciA grown in the presence (black bars) or absence (grey bars) of ATc at the indicated time points during continual log growth curves. Accompanying tables display averages ± standard deviations from data depicted in histograms, along with p-values determined by Student’s t-test. (TIF) [file pgen.1007115.s002.tif]

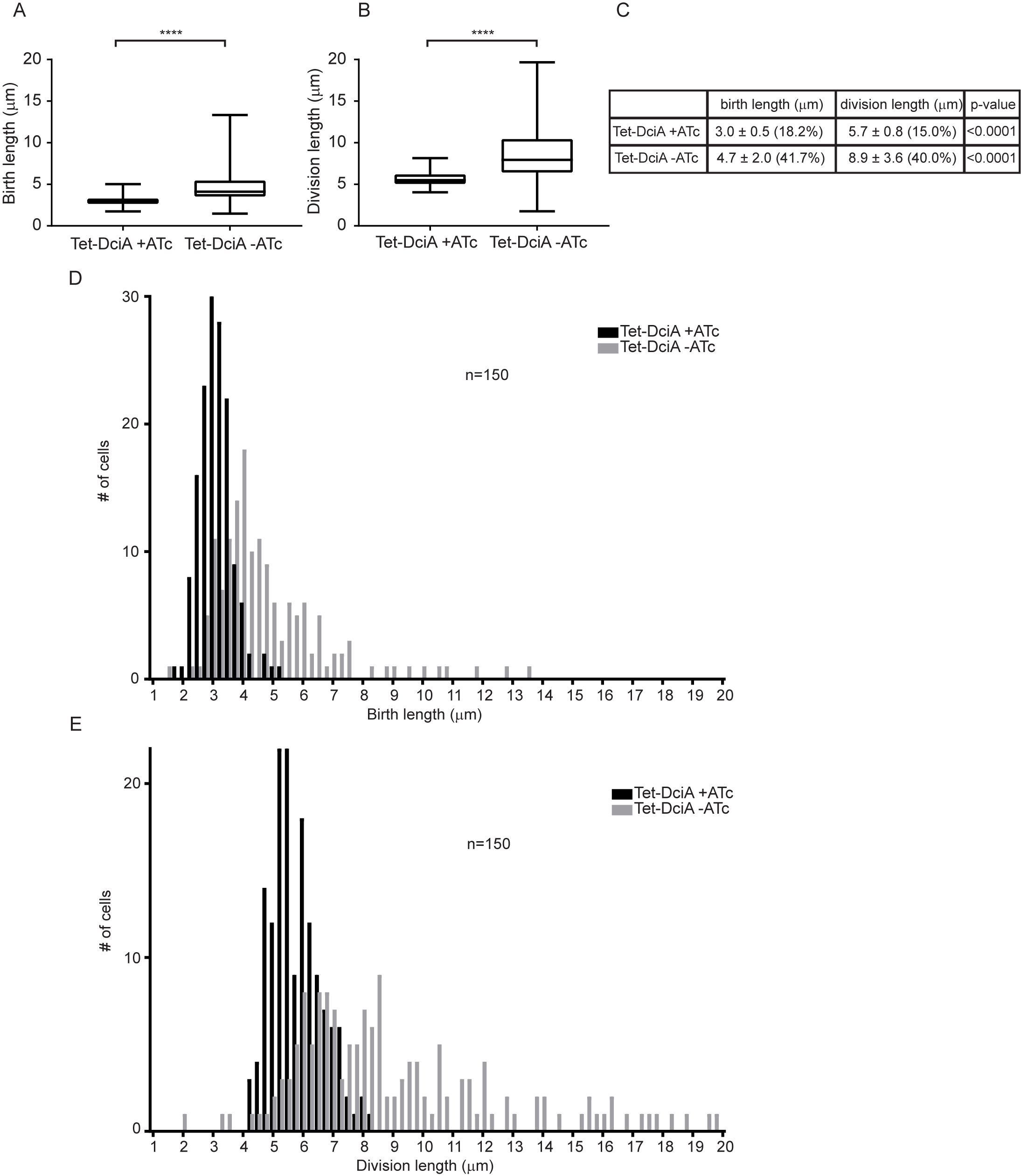

Supplement: S3 Fig — (A,B) Box plots demonstrating the median length of 150 Tet-DciA cells grown in replete (+ATc) and 150 Tet-DciA cells grown in depleting (-ATc) conditions at birth (A) and division (B). (C) Table displays mean ± SD and (coefficient of variation) from data depicted in previous box plots and subsequent histograms, along with p-values comparing median lengths determined by Student’s t-test, **** p <0.0001. (D,E) Histograms of cell length at birth (D) or division (E) of the same 150 Tet-DciA cells grown +ATc (black bars) and -ATc (grey bars). (TIF) [file pgen.1007115.s003.tif]

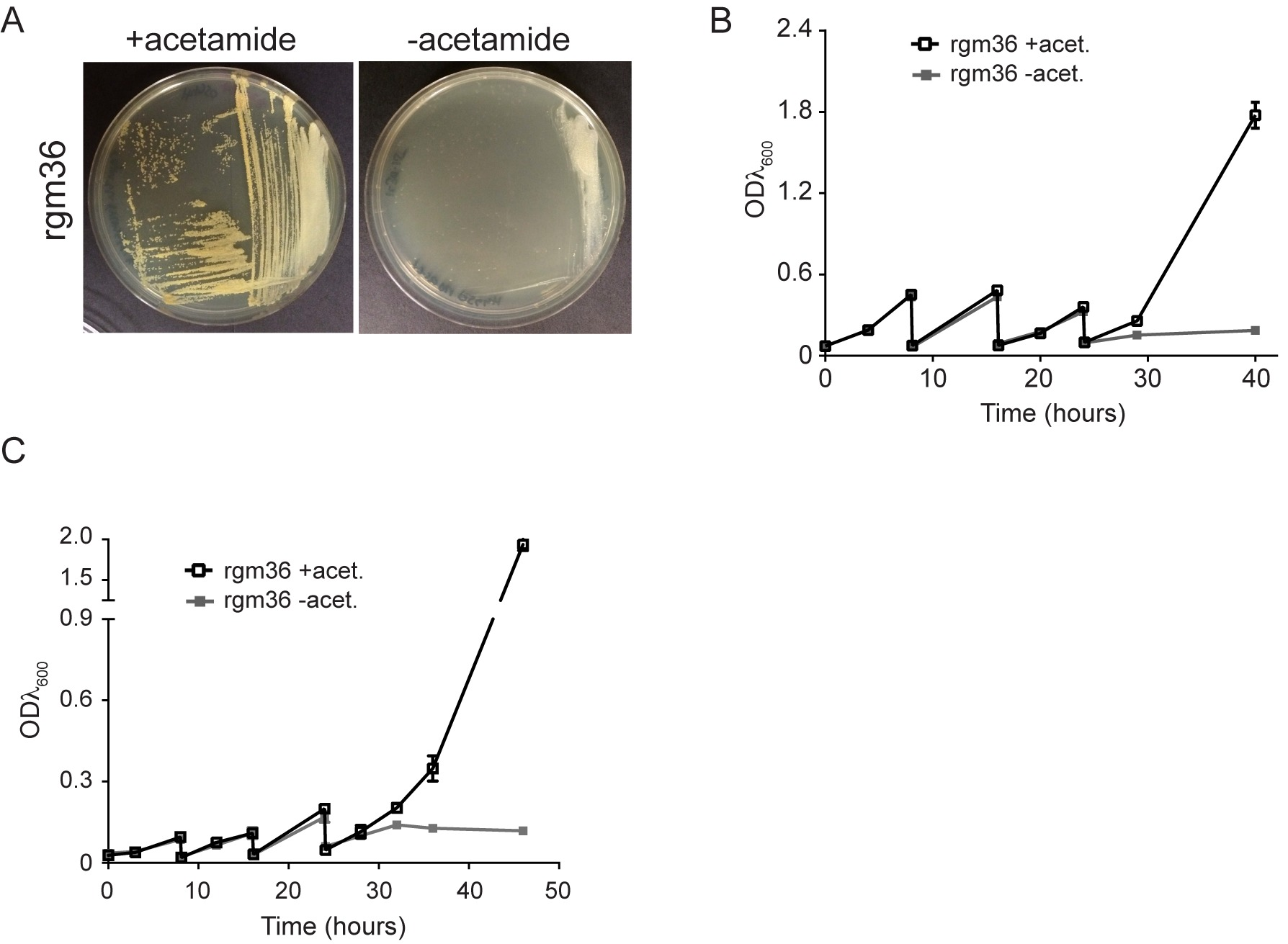

Supplement: S4 Fig — Growth of rgm36, an acetamide (acet.)-inducible dnaA depletion M. smegmatis strain (A) on plates and (B,C) in continual log growth curves with two biological replicates per condition per growth curve (total of n = 4 +acet., n = 4 –acet.). The mean ± SEM is graphed. (TIF) [file pgen.1007115.s004.tif]

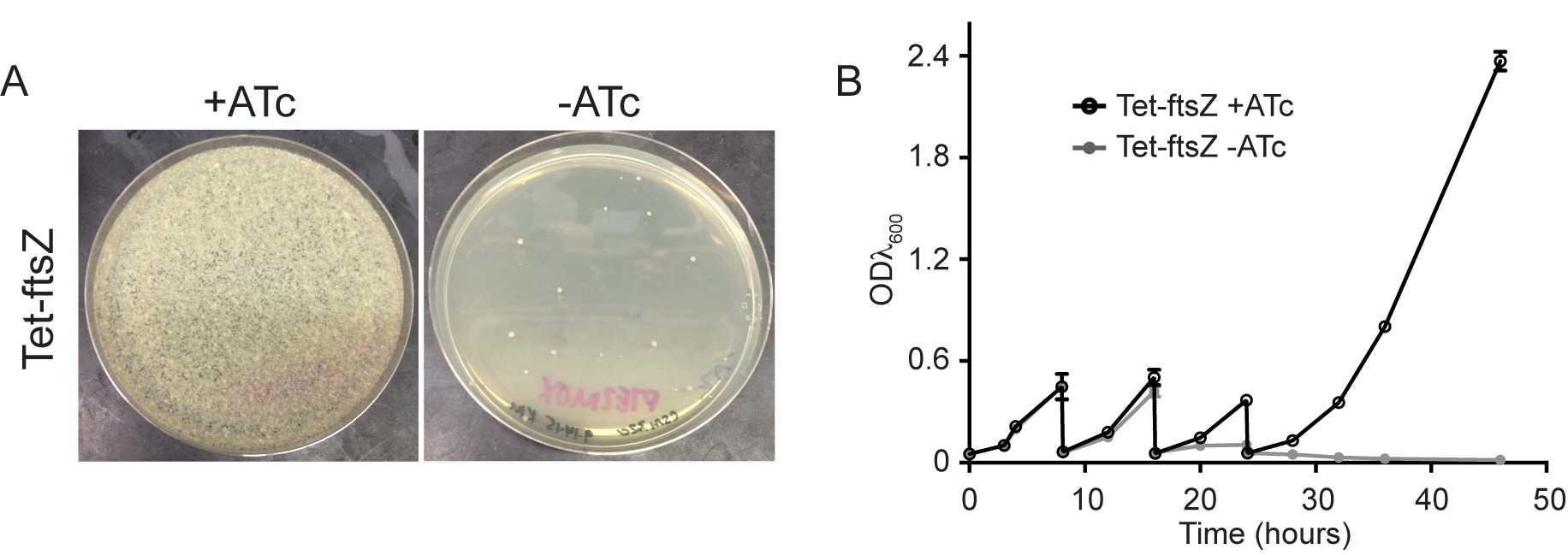

Supplement: S5 Fig — Growth of csm362, a TetOn FtsZ depletion M. smegmatis strain (A) on plates and (B) in a continual log growth curve with four biological replicates per condition. The mean ± SEM is graphed. (TIF) [file pgen.1007115.s005.tif]

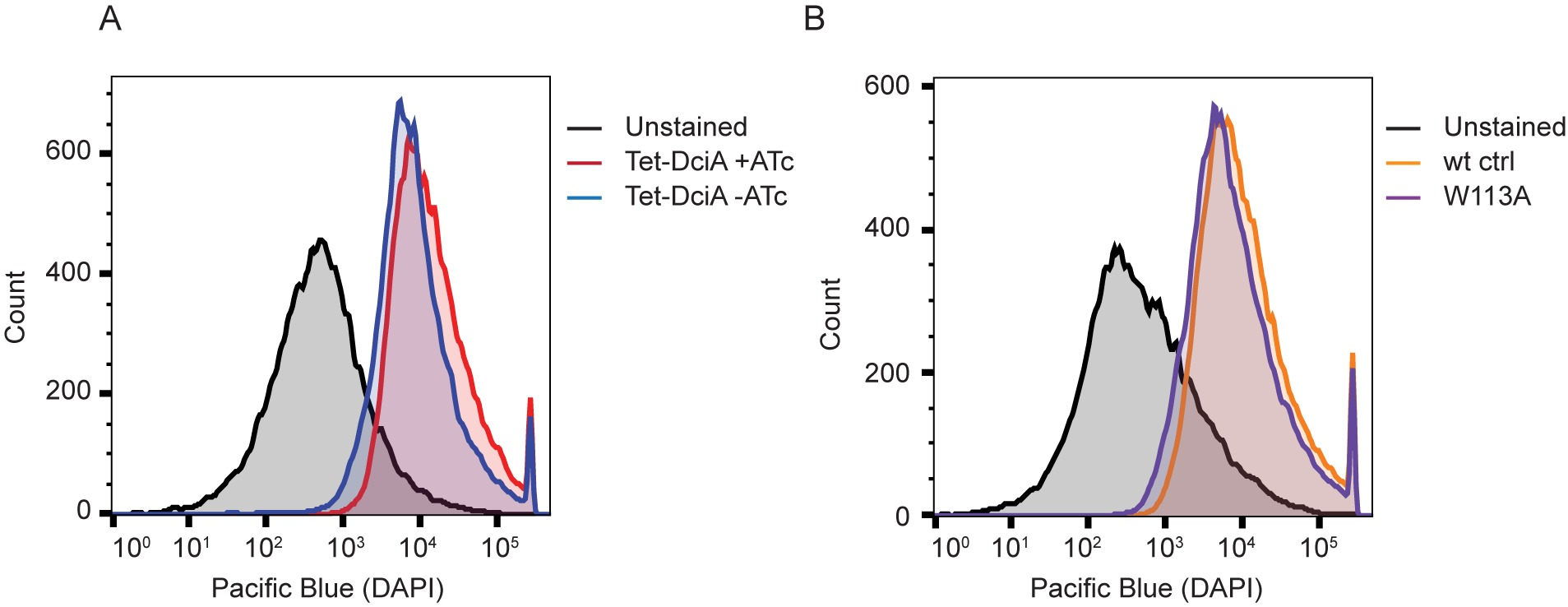

Supplement: S6 Fig — (A) Representative flow cytometry histograms in the Pacific Blue channel, which measures DAPI fluorescence, of unstained Tet-DciA cells (black), DAPI stained Tet-DciA grown in depleted (-ATc, blue), replete (+ATc, red) conditions. Cells were collected at the 36 hour time point of a continual logarithmic growth curve. Histograms like this were used to calculate DAPI mean fluorescence intensity (MFI) plotted for several biological replicates shown in Fig 5D. (B) Representative flow cytometry histograms in the Pacific Blue channel of unstained cells (black), DAPI stained wild-type control (wt ctrl, orange), and W113A (purple) cells. Histograms like this were used to calculate DAPI MFI plotted for several biological replicates shown in Fig 8G. (TIF) [file pgen.1007115.s006.tif]

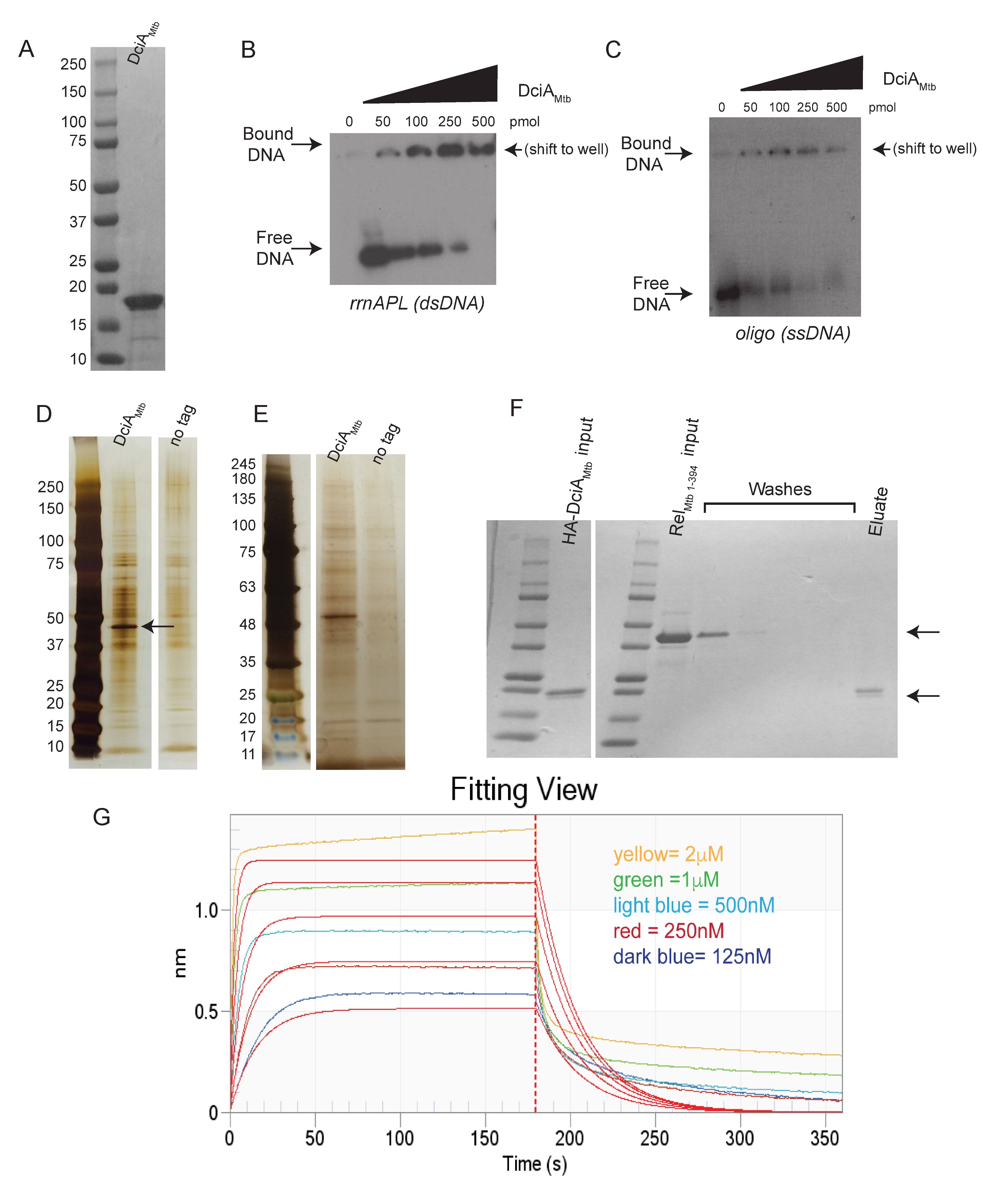

Supplement: S7 Fig — (A) GelCode Blue-stained SDS-PAGE gel of purified DciAMtb protein used in electromobility shift assays. Minor lower molecular weight protein bands were also identified as DciAMtb by mass spectrometry. (B,C) Autoradiographs of electromobility shift assay with DciAMtb protein binding 1.4ng of 32P-labeled 333 base pair rrnAPL double-stranded DNA (B) or 546pg of 32P-labeled 72 nucleotide oligo single-stranded DNA (C). All lanes contain 32P-labeled substrate DNA. Lane 1 contains no protein, while the amount of DciAMtb in the other lanes is indicated above each autoradiograph. (D,E) Silver-stained SDS-PAGE of proteins that co-immunoprecipitated with HA-DciAMtb in lysate from the M. smegmatis HA-DciAMtb or ΔdciAMsm attB::tetdciAMtb (no tag) strains. (D) Arrow indicates band containing ClpX. (E) Lysates were treated with DNase I prior to immunoprecipitation. (F) GelCode Blue-stained SDS-PAGE of purified proteins that were used as input, proteins that came off in sequential washes (see Methods), and eluate from a pull-down experiment with HA-DciAMtb as bait and RelMtb1-394 as prey. Top arrow indicates size of RelMtb1-394, bottom arrow indicates size of HA-DciAMtb. (G) The yellow, green, light blue, red, and dark blue curves are the same representative curves shown in Fig 6E of the association and dissociation of DciAMtb at the indicated concentrations to and from biotinylated-DnaB as measured by biolayer interferometry. The red lines are global fits for each corresponding concentration of DciAMtb calculated by ForteBio Data Analysis 6.4 software based on a 1:1 kinetic binding model. (TIF) [file pgen.1007115.s007.tif]

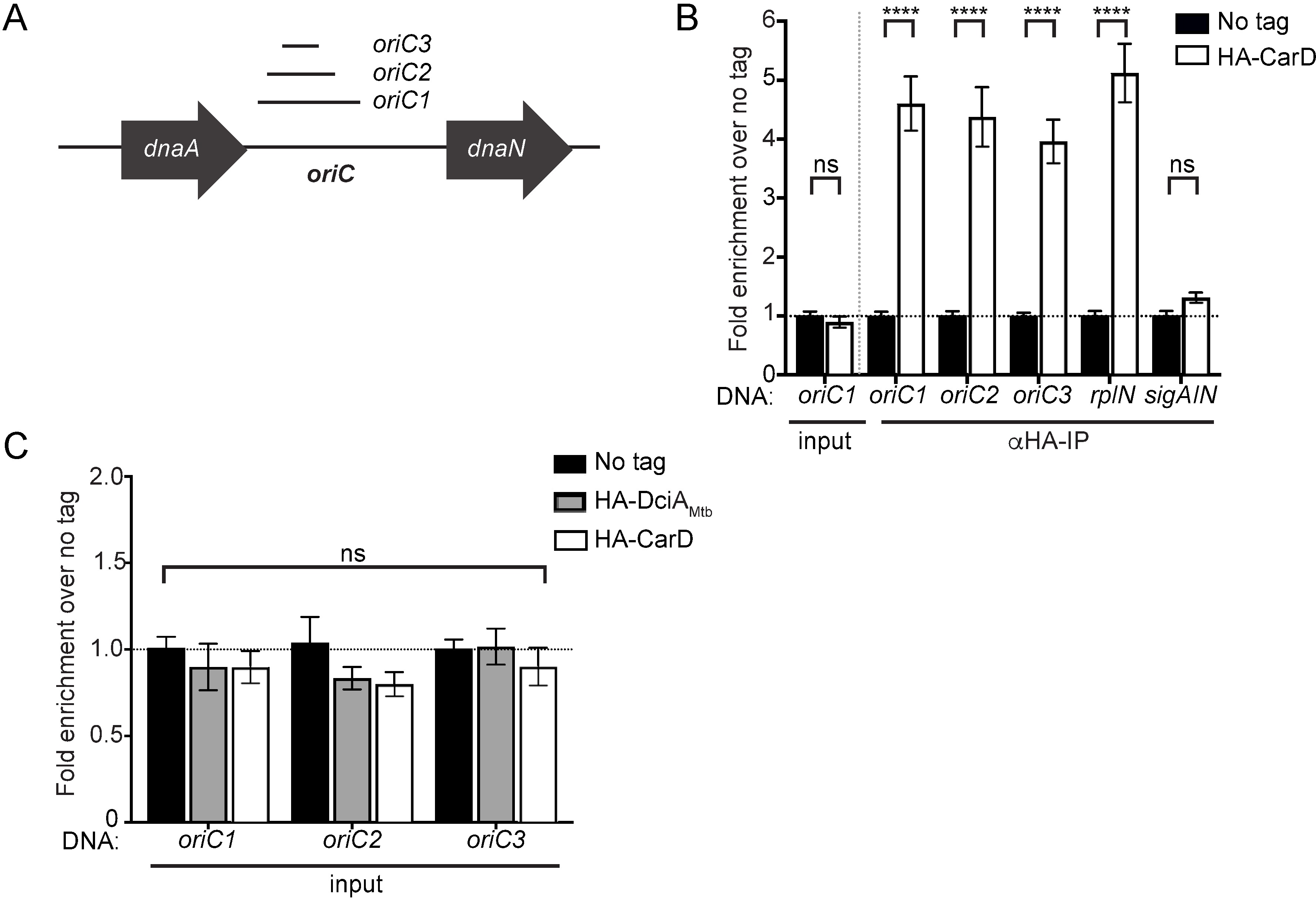

Supplement: S8 Fig — (A) Schematic of DNA fragments generated by PCR using ori1, ori2, and ori3 primers. (B) Fold enrichment in DNA fragments that co-immunoprecipitated with HA-CarD (αHA-IP) or are present in input samples (input) relative to the No tag strain. Bars represent mean ± SEM (n = 5 except No tag sigAIN, which is n = 4). (C) Fold enrichment in levels of DNA fragments containing oriC (oriC1-3) in input (non-immunoprecipitated) samples of lysates from strains expressing untagged DciAMtb (No tag, solid black bars), HA-DciAMtb (grey bars), and HA-CarD (white bars) strains. Data is represented as fold enrichment relative to No tag levels and shows that none of these DNA fragments were enriched prior to immunoprecipitation. **** p <0.0001, ns is not significant, statistical significance was determined by one-way ANOVA and Tukey’s multiple comparison test. (TIF) [file pgen.1007115.s008.tif]

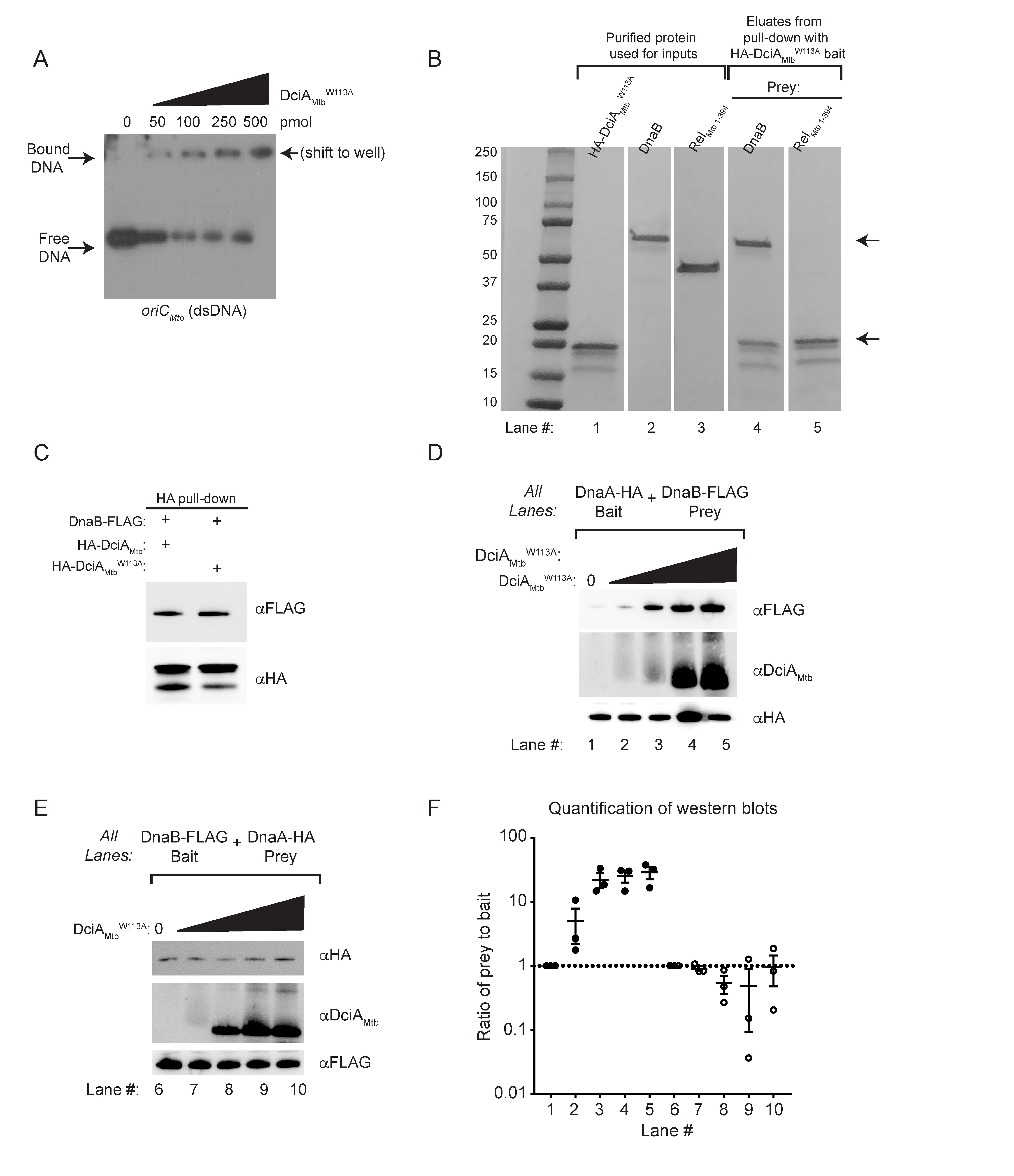

Supplement: S9 Fig — (A) Autoradiograph of EMSA with DciAMtbW113A protein and 4.8 ng oriCMtb dsDNA separated by native PAGE. All lanes contain 32P-labeled oriCMtb DNA. Amount of DciAMtbW113A in each lane is indicated. (B) GelCode Blue-stained SDS-PAGE of purified proteins that were used for inputs and eluates from pull-down experiments with DciAMtbW113A as bait and the indicated protein as prey. These proteins were not treated with Benzonase during purification. Top arrow indicates approximate sizes of DnaB and RelMtb1-394, bottom arrow indicates size of DciAMtbW113A. (C) Western blot analysis of pull-downs with either HA- DciAMtb or HA- DciAMtbW113A as bait and DnaB-FLAG as prey. Proteins were treated with Benzonase during purification (D,E) Representative western blot of pull-downs with (D) DnaA-HA as bait and DnaB-FLAG as prey or (E) DnaB-FLAG as bait and DnaA-HA as prey and either no (lanes 1,6), 0.5x (lanes 2,7), 1x (lanes 3,8), 2x (lanes 4,9), or 4x (lanes 5,10) molar ratio of DciAMtbW113A relative to the bait. (F) Quantification of the ratio of prey-to-bait for triplicate western blots like those shown in (D) and (E) where the ratio for lanes with no DciAMtbW113A is set to 1 and the ratio for all other samples is relative to the lane with no DciAMtbW113A. Symbols represent each replicate, center values and error bars represent mean ± SEM. (TIF) [file pgen.1007115.s009.tif]
