# Supplementary material for: Rv0004 is a new essential member of the mycobacterial DNA replication machinery
Source: PLoS Genet. 2017 Nov 27;13(11):e1007115. doi: 10.1371/journal.pgen.1007115 (PMC5720831; doi:10.1371/journal.pgen.1007115)
Supplement: S1 Table — (DOCX) [file pgen.1007115.s010.docx]

**Supplemental Table S1. Bacterial strains used in this study.**

| **Name** | **Bacteria** | **Description** | **Use** | **Source** |
| --- | --- | --- | --- | --- |
| Mtb Erdman | *M. tuberculosis* | Wild-type | RNA extraction | Lab strain |
| Mc^2^155 | *M. smegmatis* | Wild-type | RNA extraction; used to create Δ*dciA_Msm_ attB::*tet*dciA_Mtb_* | Lab strain |
| Δ*dciA_Msm_ attB::*tet*dciA_Mtb_* | Strain (*M. smegmatis)* | Endogenous dciA_Msm_ (*MSMEG_0004)* deleted; dciA_Mtb_ (Erdman_0004) integrated at *attB* site by using pMSG430 (*kan^R^*) | Backbone to create other strains through allelic exchange; | This study |
| Tet-DciA | *M. smegmatis* | Δ*dciA_Msm_ attB::*tet*dciA_Mtb_* transformed with pTetR (*hyg^R^*) | Tet-On *dciA_Mtb_* depletion strain | This study |
| rgm36 | *M. smegmatis* | Endogenous *dnaA* deleted; dnaA under the control of the amylase promoter [Greendyke et al. 2002] | Acetamide-On *dnaA* depletion strain | [1] |
| csm362 | *M. smegmatis* | Endogenous ftsZ deleted; ftsZ integrated at *attB* site by using pMSG430 (*kan^R^*); transformed with pTetR (*hyg^R^*) | Tet-On *ftsZ* depletion strain | This study |
| Δ*dciA_Msm_ attB::*tet*dciA_Mtb_Zeo* | *M. smegmatis* | Endogenous dciA_Msm_ (*MSMEG_0004)* deleted; dciA_Mtb_ (Erdman_0004) integrated at *attB* site by using pDB19 (*zeo^R^*) | Served as backbone to create HA-DciA_Mtb_ and W113A strains through gene-switching/marker exchange | This study |
| HA-DciA_Mtb_ | *M. smegmatis* | Generated through the process of gene-switching/marker-exchange by transforming Δ*dciA_Msm_ attB::*tet*dciA_Mtb_zeo* with pMSG430 HA-DciA_Mtb_ | *In vivo* Immunoprecipitation experiments, as only allele of DciA_Mtb_ is HA-tagged. | This study |
| W113A | *M. smegmatis* | Generated through the process of gene-switching/marker-exchange by transforming Δ*dciA_Msm_ attB::*tet*dciA_Mtb_zeo* with pMSG430 DciA_Mtb_^W113A^ | Only allele of DciA is point mutant DciA_Mtb_^W113A^ | This study |
| Wild-type Control (wt ctrl) | *M. smegmatis* | Generated through the process of gene-switching/marker-exchange by transforming Δ*dciA_Msm_ attB::*tet*dciA_Mtb_zeo* with pMSG430 DciA_Mtb_ (genetically same as Δ*dciA_Msm_ attB::*tet*dciA_Mtb_* but made through gene switching in parallel with the W113A strain) | Control for W113A | This study |
| HA-CarD | *M. smegmatis* | Endogenous *carD_Msm_* is deleted, C-terminally HA-tagged CarD_Mtb_ is expressed from the *attB* site as the only allele of *carD* | Used as positive control for ChIP | [2] |
| DH5α | *E. coli* |  | Used for cloning | Invitrogen |
| BL21 (DE3) | *E. coli* |  | Used to induce proteins through pGEX-6P and pET-SUMO | Novagen |

**Reference**

1. Greendyke R, Rajagopalan M, Parish T, Madiraju MVVS. Conditional expression of Mycobacterium smegmatis dnaA, an essential DNA replication gene. Microbiology. 2002;148: 3887–900. Available: http://www.ncbi.nlm.nih.gov/pubmed/12480893

2. Stallings CL, Stephanou NC, Chu L, Hochschild A, Nickels BE. CarD Is an Essential Regulator of rRNA Transcription Required for Mycobacterium tuberculosis Persistence. Cell. Elsevier Ltd; 2009;138: 146–159. doi:10.1016/j.cell.2009.04.041
