# Supplementary material for: Rv0004 is a new essential member of the mycobacterial DNA replication machinery
Source: PLoS Genet. 2017 Nov 27;13(11):e1007115. doi: 10.1371/journal.pgen.1007115 (PMC5720831; doi:10.1371/journal.pgen.1007115)
Supplement: S2 Table — (DOCX) [file pgen.1007115.s011.docx]

**Supplementary Table S2. Plasmids used in this study.**

| **Name** | **Antibiotic Resistance** | **Description/Sequence** | **Use** | **Source** |
| --- | --- | --- | --- | --- |
| pMSG430 | *kan^R^* | contains *attP, tetO,* encodes integrase | To construct *dciA_Msm_ attB::*tet*dciA_Mtb_* and other strains | Described in [1,2] |
| pDB88 | *hyg^R^*, but results in unmarked deletion | Two step allelic exchange (Barkan et al. 2011) | To delete endogenous *dciA_Msm_* | [3] |
| pDB19 | *zeo^R^* | contains *attP, tetO,* encodes integrase | Gene-switching/Marker exchange | Described in [1,2] |
| pTetR | *hyg^R^* | Episomal, expresses TetOn Tet Repressor | To create depletion strains | [4] |
| pGEX-6P | *carb^R^* | Protein purification vector, encocdes N-terminal GST tag | Recombinant expression of mycobacterial proteins in BL21 | GE Healthcare Life Sciences |
| pET-SUMO | *kan^R^* | Protein purification vector, encocdes N-terminal His-SUMO tag | Recombinant expression of mycobacterial proteins in BL21 | Invitrogen |

**References**

1. Garner AL, Weiss LA, Manzano AR, Galburt EA, Stallings CL. CarD integrates three functional modules to promote efficient transcription, antibiotic tolerance, and pathogenesis in mycobacteria. Mol Microbiol. 2014;93: 682–97. doi:10.1111/mmi.12681

2. Stallings CL, Stephanou NC, Chu L, Hochschild A, Nickels BE. CarD Is an Essential Regulator of rRNA Transcription Required for Mycobacterium tuberculosis Persistence. Cell. Elsevier Ltd; 2009;138: 146–159. doi:10.1016/j.cell.2009.04.041

3. Barkan D, Stallings CL, Glickman MS. An improved counterselectable marker system for mycobacterial recombination using galK and 2-deoxy-galactose. Gene. 2011;470: 31–36. doi:10.1016/j.gene.2010.09.005

4. Ehrt S, Guo X V, Hickey CM, Ryou M, Monteleone M, Riley LW, et al. Controlling gene expression in mycobacteria with anhydrotetracycline and Tet repressor. Nucleic Acids Res. Oxford University Press; 2005;33: e21. doi:10.1093/nar/gni013
