# Supplementary material for: Rv0004 is a new essential member of the mycobacterial DNA replication machinery
Source: PLoS Genet. 2017 Nov 27;13(11):e1007115. doi: 10.1371/journal.pgen.1007115 (PMC5720831; doi:10.1371/journal.pgen.1007115)
Supplement: S3 Table — (DOCX) [file pgen.1007115.s012.docx]

**Supplementary Table S3. Primers used in this study.**

| **Name** | **Sequence** | **Use** | **Source** |
| --- | --- | --- | --- |
| *dciA_Msm_ (MSMEG_0004)* | TCACCCAGGACCTCGCGAAATC | qRT-PCR For | This study |
| *dciA_Msm_ (MSMEG_0004)* | CAGCACGCCTTCGTTGAGC | qRT-PCR Rev | This study |
| *dciA_Mtb_ (Mtb_0004)* | CTGGCAAAGAAACGCGGCTG | qRT-PCR For | This study |
| *dciA_Mtb_ (Mtb_0004)* | TCAACACCCCGTCGTTTAGCG | qRT-PCR Rev | This study |
| *M. smeg_*SigA | TGCCGATCTGCTTGAGGTAGG | qRT-PCR For, ChIP-qPCR For | [1] |
| *M. smeg_*SigA | CTTCGTGTGGGACGAGGAAGAG | qRT-PCR Rev, ChIP-qPCR Rev | [1] |
| M. smeg 16S rRNA | GTGCATGTCAAACCCAGGTAAGG | qRT-PCR For | [1] |
| M. smeg 16S rRNA | GGGATCCGTGCCGTAGCTAAC | qRT-PCR Rev | [1] |
| Mtb 16S rRNA | GAGATACTCGAGTGGCGAAC | qRT-PCR For | [1] |
| Mtb 16S rRNA | GGCCGGCTACCCGTCGTC | qRT-PCR Rev | [1] |
| *MSMEG_dnaA* | AGGAGGAGTTCTTCCACACCTTC | qRT-PCR For | This study |
| *MSMEG_dnaA* | GCTGGACATCGGTGATGAGGC | qRT-PCR Rev | This study |
| *MSMEG_dnaN* | CGACCGACCGGTTCCGTCT | qRT-PCR For | This study |
| *MSMEG_dnaN* | GCCAGATGCACCTGGTTGCC | qRT-PCR Rev | This study |
| *MSMEG_0002* | TGGGTGCTGGCCACTACG | qRT-PCR For | This study |
| *MSMEG_0002* | GCCTGCGGATCCTTGATGAGTTC | qRT-PCR Rev | This study |
| *MSMEG_recF* | CCGCTGCGGGAGCACGTTA | qRT-PCR For | This study |
| *MSMEG_recF* | CGGTGCGAGCAACTGGTAGG | qRT-PCR Rev | This study |
| *MSMEG_gyrB* | GTTCGAGGGCCAGACCAAGAC | qRT-PCR For | This study |
| *MSMEG_gyrB* | GCCGACGAGACCGCTTTGTTG | qRT-PCR Rev | This study |
| *Mtb* oriC For | GCATCCGTCAGCGCTCCAAG | To amplify EMSA substrate *oriC_Mtb_* | [2] |
| *Mtb* oriC Rev | TGCGCCCTTTCACCTCACGATG | To amplify EMSA substrate *oriC_Mtb_* | [2] |
| *M.smeg* rrnAPL For | AGAGGCGGACAAAAAACAACAAACAAAAAC | To amplify EMSA substrate *rrnAPL* | [3] |
| *M.smeg* rrnAPL Rev | TCCGTTGTTCGTGGAAAACCTGG | To amplify EMSA substrate *rrnAPL* | [3] |
| 3x Flag Rev | CTAGCCTTGTCATCGTCATCCTTGTAATCGATGTCATGATCTTTATAATCACCGTCATGGTCTTTGTAGTCGGACATG | Used as EMSA substrate 3xFlag | This study |
| *oriC1* For | GTGAGCAACTTCTGTCACACGC | ChIP-qPCR For | This study |
| *oriC1* Rev | GATATTCGAGTAACAGTAATAGGTGCTGTGG | ChIP-qPCR Rev | This study |
| *oriC2* For | GCTCGGCGGCTGTGGATACC | ChIP-qPCR For | This study |
| *oriC2* Rev | GAACGCCTGTTTGCCCAGCATC | ChIP-qPCR Rev | This study |
| *oriC3* For | CGCGCAAACCACAGGATGAC | ChIP-qPCR For | This study |
| *oriC3* Rev | GTCGCGGTGTGAGTGGTGTGT | ChIP-qPCR Rev | This study |
| *rplN* For | GATCGCGCTGAGTTCACACTCG | ChIP-qPCR For | [1] |
| *rplN* Rev | GATCTCCTGACCTGGTTTTGTATGCAC | ChIP-qPCR Rev | [1] |
| Msmeg_0004 5’forward XbaI | GTCTAGAGCGCACGGCCTTGCTGAAAA | To amplify upstream homology of *MSMEG_0004 (dciA_Msm_)*region for pDB88 (Forward) | This study |
| MSMEG_0004 5’reverseNdeI | GCATATGGGTCATGAGGCCACCATCGAAAC | To amplify upstream homology of *MSMEG_0004 (dciA_Msm_)*region for pDB88 (Reverse) | This study |
| MSMEG_0004 3’forwardNdeI | GCATATGTGAGGCGTGTCACACCTCTTG | To amplify downstream homology of *MSMEG_0004 (dciA_Msm_)*region for pDB88 (Forward) | This study |
| MSMEG_0004 3’reverseScaI | GAGTACTGCAGCTGGGTCATGACGACATC | To amplify downstream homology of *MSMEG_0004 (dciA_Msm_)*region for pDB88 (Reverse) | This study |
| MtbErdman_0004 EcoRI For | GGAATTCATGAAGTCACCAGGGTTGGATTTGGTC | To amplify Rv*0004* (*dciA_Mtb_*)for pMSG430 (Forward) | This study |
| MtbErdman_0004 HinDIII rev | GAAGCTTTTCTGGGCCGATCGACGTGTTA | To amplify *Rv0004 (dciA_Mtb_),* HA-Rv0004 (HA-DciA_Mtb_), and Rv0004-W113A (DciA_Mtb_^W113A^) for pMSG430 (Reverse) | This study |
| Erdman0004 GLDL NtermHA For EcoRI | GGAATTCATGTACCCATACGATGTTCCTGACTATGCGGGGTTGGATTTGGTCAGGCG | To amplify HA-Rv0004 (HA-DciA_Mtb_) into pMSG430 (Forward) | This study |
| MtbErdman_0004 BamHI For | GGGATCCATGAAGTCACCAGGGTTGGATTTGGTC | To amplify Rv0004 for pGEX-6P (Forward) | This study |
| erd0004GLDL For BamHI | GGGATCCGGGTTGGATTTGGTCAGGCGCA | To amplify Rv0004^W113A^ (DciA_Mtb_^W113A^) for pGEX (Forward) | This study |
| MtbErdman_0004 HinDIII Rev | CAAGCTTTTCTGGGCCGATCGACGTGTTA | To amplify Rv0004 (DciA_Mtb_) and HA-Rv0004 (HA-DciA_Mtb_) for pGEX-6P (Reverse) | This study |
| N-HA gldl erd0004 BamHI | GGGATCCTACCCATACGATGTTCCTGACTATGCGG | To amplify HA-Rv0004 (HA-DciA_Mtb_), HA-Rv0004^W113A^ (HA-DciA_Mtb_^W113A^), and HA-Rv0004 (HA-DciA_Mtb_) N-terminus for pGEX-6P (Forward) | This study |
| Mtb DnaA For BamHI | GGGATCCTTGACCGATGACCCCGGTTC | To amplify *Mtb* DnaA for pGEX-6P (Forward) | This study |
| Mtb DnaA Rev NotI | GGCGGCCGCGCCGTGCTAGCGCTTGGAG | To amplify *Mtb* DnaA for pGEX-6P (Reverse) | This study |
| DnaA Rev HA stop NotI | GGCGGCCGCTTACGCATAGTCAGGAACATCGTATGGGTAGCGCTTGGAGCGCTGAC | To amplify *Mtb* DnaA-HA for pGEX-6P (Reverse) | This study |
| DnaB Nterm For BamHI | GGGATCCATGGCGGTCGTTGATGACCTAGC | To amplify *Mtb* DnaB for pET-SUMO (Forward) | This study |
| dnaB Cterm Rev HinDIII | GAAGCTTTCACCGAGCCATGTTGGCGAAG | To amplify *Mtb* DnaB for pET-SUMO (Reverse) | This study |
| DnaB Rev Flagstop XhoI | GCTCGAGTTACTTGTCGTCATCGTCTTTGTAGTCCCGAGCCATGTTGGCGAAGC | To amplify *Mtb* DnaB-Flag for pET-SUMO (Reverse) | This study |
| dnaB nterm Rev | TCGGCATCTTGTTCCAGCGAGCCCGATTCCCTGAGGTCG | Overlap PCR primer to amplify DnaB Nterminus to get rid of intein (Rev) | This study |
| dnaB Cterm For actual | GCCGACCTCAGGGAATCGGGCTCGCTGGAACAAGATGCCGA | Overlap PCR primer to amplify DnaB Cterminus to get rid of intein (For) | This study |
| STAWAT WtoA erd0004 | TCGACGGCGGCGGCGACGCAGTTG | Overlap PCR primer to generate W113A allele (Forward) | This study |
| Revcomp_ STAWAT WtoA erd0004 | CAACTGCGTCGCCGCCGCCGTCGA | Overlap PCR primer to generate W113A allele (Reverse) | This study |
| Erdman0004 GLDL For EcoRI | GGAATTCGGGTTGGATTTGGTCAGGCG | To amplify Rv0004^W113A^ for pMSG430 (Forward) | This study |
| Smeg FtsZ For XbaI | GTCTAGAATGACCCCCCCGCATAACTACCTC | To amplify *M. smegmatis* *ftsZ* for pMSG430 (Forward) | This study |
| Smeg FtsZ Rev EcorI | GGAATTCGCTCAGTGCCGCATGAAGGG | To amplify *M. smegmatis* *ftsZ* for pMSG430 (Reverse) | This study |
| Msm ftsZ upstream For | GCATATGACCTTGATGACCGCGAGGTAGTT | To amplify upstream homology region of *M. smegmatis* *ftsZ* for pDB88 (Forward) | This study |
| Msm ftsZ upstream Rev SspI | GAATATTCGTGGTGGTCAAGGACTATCCG | To amplify upstream homology region of *M. smegmatis* *ftsZ* for pDB88 (Reverse) | This study |
| Msm ftsZ downstream For XbaI | GTCTAGACCCAAACCCTTTAATTGACGGACGATTC | To amplify downstream homology region of *M. smegmatis* *ftsZ* for pDB88 (Forward) | This study |
| Msm ftsZ downstream Rev NdeI | GCATATGGCCCTTCATGCGGCACTGAG | To amplify downstream homology region of *M. smegmatis* *ftsZ* for pDB88 (Reverse) | This study |
| Erdman0004 Nterm +stop Rev SalI | GGTCGACTTAGTGGCCGACCACCGCAG | To amplify Rv0004 (DciA_Mtb_) N-terminus for pGEX-6P | This study |
| Erdman0004 HA-Cterminus For BamHI | GGGATCCTACCCATACGATGTTCCTGACTATGCGCAGATCGCCGAACATGCACGC | To amplify Rv0004 (DciA_Mtb_) and Rv0004-W113A (DciA_Mtb_^W113A^) C-terminus for pGEX-6P | This study |

**References:**

1. Stallings CL, Stephanou NC, Chu L, Hochschild A, Nickels BE. CarD Is an Essential Regulator of rRNA Transcription Required for Mycobacterium tuberculosis Persistence. Cell. Elsevier Ltd; 2009;138: 146–159. doi:10.1016/j.cell.2009.04.041

2. Madiraju MVVS, Moomey M, Neuenschwander PF, Muniruzzaman S, Yamamoto K, Grimwade JE, et al. The intrinsic ATPase activity of Mycobacterium tuberculosis DnaA promotes rapid oligomerization of DnaA on oriC. Mol Microbiol. 2006;59: 1876–90. doi:10.1111/j.1365-2958.2006.05068.x

3. Garner AL, Weiss LA, Manzano AR, Galburt EA, Stallings CL. CarD integrates three functional modules to promote efficient transcription, antibiotic tolerance, and pathogenesis in mycobacteria. Mol Microbiol. 2014;93: 682–97. doi:10.1111/mmi.12681
